# Bayesian group sequential designs for phase III emergency medicine trials: a case study using the PARAMEDIC2 trial

**DOI:** 10.1186/s13063-019-4024-x

**Published:** 2020-01-14

**Authors:** Elizabeth G. Ryan, Nigel Stallard, Ranjit Lall, Chen Ji, Gavin D. Perkins, Simon Gates

**Affiliations:** 1 Cancer Research UK Clinical Trials Unit, Institute of Cancer and Genomic Sciences, University of Birmingham, Birmingham, B15 2TT UK; 2 Warwick Clinical Trials Unit, Warwick Medical School, University of Warwick, Coventry, CV4 7AL UK; 3 Statistics and Epidemiology, Division of Health Sciences, Warwick Medical School, University of Warwick, Coventry, CV4 7AL UK; 4 Critical Care Directorate, University Hospitals Birmingham NHS Foundation Trust, Birmingham, B15 2TT UK

**Keywords:** Bayesian design, Group sequential design, Interim analysis, Cardiac arrest, Randomised controlled trials, Monitoring, Phase III

## Abstract

**Background:**

Phase III trials often require large sample sizes, leading to high costs and delays in clinical decision-making. Group sequential designs can improve trial efficiency by allowing for early stopping for efficacy and/or futility and thus may decrease the sample size, trial duration and associated costs. Bayesian approaches may offer additional benefits by incorporating previous information into the analyses and using decision criteria that are more practically relevant than those used in frequentist approaches. Frequentist group sequential designs have often been used for phase III studies, but the use of Bayesian group sequential designs is less common. The aim of this work was to explore how Bayesian group sequential designs could be constructed for phase III trials conducted in emergency medicine.

**Methods:**

The PARAMEDIC2 trial was a phase III randomised controlled trial that compared the use of adrenaline to placebo in out-of-hospital cardiac arrest patients on 30-day survival rates. It used a frequentist group sequential design to allow early stopping for efficacy or harm. We constructed several alternative Bayesian group sequential designs and studied their operating characteristics via simulation. We then virtually re-executed the trial by applying the Bayesian designs to the PARAMEDIC2 data to demonstrate what might have happened if these designs had been used in practice.

**Results:**

We produced three alternative Bayesian group sequential designs, each of which had greater than 90% power to detect the target treatment effect. A Bayesian design which performed interim analyses every 500 patients recruited produced the lowest average sample size. Using the alternative designs, the PARAMEDIC2 trial could have declared adrenaline superior for 30-day survival with approximately 1500 fewer patients.

**Conclusions:**

Using the PARAMEDIC2 trial as a case study, we demonstrated how Bayesian group sequential designs can be constructed for phase III emergency medicine trials. The Bayesian framework enabled us to obtain efficient designs using decision criteria based on the probability of benefit or harm. It also enabled us to incorporate information from previous studies on the treatment effect via the prior distributions. We recommend the wider use of Bayesian approaches in phase III clinical trials.

**Trial registration:**

PARAMEDIC2 Trial registration ISRCTN, ISRCTN73485024. Registered 13 March 2014, http://www.isrctn.com/ISRCTN73485024

## Introduction

Group sequential designs, a class of adaptive design, can offer a more efficient approach than traditional fixed sample size designs for phase III randomised controlled trials (RCTs), which often use large sample sizes and require many months or years to recruit patients (e.g. [1]). These designs incorporate planned interim analyses and enable the trial to terminate early if sufficient evidence exists to reach a firm conclusion, indicated by the crossing of stopping boundaries. Trials may be stopped as soon as efficacy is established, or they may be terminated for futility. The conditions for stopping the trial must be pre-specified based on the input of the key stakeholders (e.g. clinical investigators, trial statisticians, health economists and patients) to maintain integrity and credibility of the trial. Since these trials have the opportunity to stop earlier than fixed designs, their expected sample size is smaller, leading to the potential for reduced costs. However, if group sequential designs do not stop early they may result in an increased achieved/maximum sample size, cost and trial duration compared to fixed designs for the same level of power and type I error.

Many of the phase III RCTs that have used group sequential designs have been constructed using the frequentist approach (e.g. [2, 3]). These methods have typically involved null hypothesis testing at each interim analysis and calibrating the stopping boundaries over the interim analyses to preserve an overall type I error rate of, say, 5% [1].

Bayesian statistical methods provide an alternative approach to frequentist methods and are well-suited to performing interim analyses since they were developed to combine new data with previous information or beliefs to give updated probabilities about the quantity of interest, such as the treatment effect. In the Bayesian approach, historical information or clinical opinion driven by evidence can be translated into a *prior distribution* for the treatment effect. The prior is updated with accumulated trial data to become a *posterior distribution* for the treatment effect. From the posterior distribution one can obtain the probability of the treatment effect taking various values (e.g. probability relative risk (RR) < 1).

The posterior distribution can be used at interim analyses to drive decisions, such as whether to stop for efficacy based on the probability of superiority of the intervention or the probability of a clinically significant difference. Thus, the Bayesian approach can provide clinically relevant decision criteria for the interim analyses. *See* Berry et al. [4] for additional discussion on the advantages of Bayesian adaptive designs for clinical trials.

The United States Food and Drug Administration (FDA) has provided guidance on the use of Bayesian designs for RCTs [5, 6]. Whilst Bayesian adaptive designs are increasingly being used in early phase trials, they have not been widely adopted in practice for phase III trials. Only a few published, completed phase III trials have used Bayesian adaptive methods from the design phase (e.g. [7–9]). A recent example of a phase III Bayesian group sequential design is the UK-REBOA trial [10], which is being conducted in trauma patients and currently is recruiting. Some of the reasons for the lack of uptake of Bayesian adaptive designs include the mathematical complexity introduced by some Bayesian designs and the potentially high computational cost to simulate designs and perform analysis; a lack of knowledge and skills in Bayesian adaptive trial methodology compared to traditional methods; nervousness from researchers regarding unfamiliar methods; and the requirement of having to specify a prior distribution [10]. Difficulties may also exist in obtaining funding as grant-awarders often prefer more conservative methods.

The aim of this paper is to explore in detail how a Bayesian group sequential approach could be used to design a phase III emergency medicine trial. We will use a large, recently published RCT [11, 12] that was conducted on out-of-hospital cardiac arrest (OHCA) patients to demonstrate how Bayesian group sequential designs could be constructed in this context. We will propose several Bayesian designs and compare different design features to illustrate the process by which a design might be selected. We will also perform virtual re-executions by applying these designs to the trial data and determine whether any of these designs might have led to earlier stopping in this trial. Through this work, we hope to publicise Bayesian adaptive design methods and demonstrate that they can be applied relatively easily.

## Methods

### Case study – PARAMEDIC2

The Prehospital Assessment of the Role of Adrenaline: Measuring the Effectiveness of Drug administration In Cardiac arrest study (PARAMEDIC2) was a randomised, placebo-controlled trial which investigated the effectiveness of standard of care adrenaline (epinephrine) administered by paramedics to patients who had an OHCA in the United Kingdom [11, 12]. The primary outcome was status of survival at 30 days. The aim of the PARAMEDIC2 trial was to investigate whether use of placebo improved long-term survival rates as it was thought that adrenaline may be harmful.

The planned sample size was 8000 patients, and the trial was designed using frequentist group sequential methods. The original study had 93% power to detect a difference corresponding to 8% 30-day survival in the adrenaline group relative to 6% in the placebo group, that is, a RR of 1.33, using a two-sided significance level of 0.05. An assumption of very little missing data was made for the primary outcome, and therefore, the sample size was not adjusted to account for missing data.

PARAMEDIC2 had pre-specified up to ten 3-monthly interim analyses that were performed on the 30-day survival rate. These interim analyses enabled early efficacy stopping to declare adrenaline superior, or stopping for adrenaline being harmful (placebo superior). A higher level of evidence was required in the earlier interim analyses to stop for concluding that adrenaline was harmful (placebo superior) since this would involve recommending the withdrawal of standard treatment based on data from a small number of patients. The investigating clinicians felt that the clinical community would be more accepting of a trial that stopped early to declare adrenaline superior, based on the existing evidence. Therefore, asymmetric stopping boundaries were implemented, where Pocock’s alpha-spending function was used to construct the boundaries for stopping for adrenaline being superior, and the O’Brien and Fleming alpha-spending function, which gives more stringent boundaries at early interim analyses, was used to construct the boundaries for stopping for adrenaline being worse than placebo (adrenaline harmful) (*see* [13]). These stopping boundaries ensured that the total type I error was 2.5% for declaring adrenaline superior, and 2.5% for declaring adrenaline worse than placebo. The spending functions were specified at the design stage.

Interim monitoring was based on the chi-squared test statistic, and nominal *p*-values were calculated and compared with the boundary values. The *p*-values associated with the chi-squared stopping boundaries for the predicted information that was originally assumed for each interim analysis are presented in Additional file 1: Table A1.1.

The PARAMEDIC2 study had slower recruitment and lower survival rates than originally anticipated, which led to the interim analyses being conducted with fewer patients than intended. The stopping boundaries were adjusted during the trial for each interim analysis to reflect the fact that less information was available than originally planned; the function to calculate the stopping boundaries was pre-specified, but the specific stopping boundary values for each interim analysis were calculated from the amount of information available at that time.

This adjustment had some undesirable consequences, in terms of stopping early for efficacy. First, because the timing of the interim analyses was specified by time rather than number of patients recruited [11, 12], most of the interim analyses were conducted early in the trial (*see* Additional file 1) with relatively small amounts of information. Whilst early looks were useful on safety grounds, the restriction to 10 interim analyses meant that because of the intensive monitoring in the early part of recruitment, the trial missed opportunities to stop for efficacy later on. In addition, the low information content of the interim analyses led to stopping boundaries that were very stringent, particularly for stopping for the placebo being superior. The *p*-values associated with the adjusted stopping boundaries are given in Additional file 1: Table A1.2. These meant that the trial would only have stopped early, especially for superiority of placebo, if a massive difference was observed between the arms.

PARAMEDIC2 randomised 8014 patients: 4015 to adrenaline and 3999 to placebo. None of the interim analyses recommended early stopping (*see* Additional file 1: Table A1.2). At 30 days post-randomisation 130/4012 (3.2%) adrenaline patients and 94/3995 (2.4%) placebo patients were alive at the final analysis. This gave an unadjusted odds ratio of 1.39 (95% CI: 1.06, 1.82) and *p* = 0.02. A Bayesian analysis performed on these data found a posterior probability of 0.99 that adrenaline was superior to placebo. The authors concluded that the use of adrenaline resulted in a significantly higher rate of 30-day survival compared to placebo [12].

### Alternative Bayesian designs

We are interested in investigating how a Bayesian approach could have been used to construct alternative designs for the PARAMEDIC2 trial and determine whether this trial could have stopped earlier if a different design had been used. The Bayesian approach will use different decision criteria and different stopping boundaries and will incorporate prior distributions. We will also explore Bayesian designs that used interim analysis schedules that differ from the original design.

To make the design process as realistic as possible, the Bayesian designs were developed by a statistician (EGR) who was independent of the PARAMEDIC2 trial, using the PARAMEDIC2 trial protocol and Statistical Analysis Plan, but without use of the observed data, to obtain trial design parameters. Discussions were held with the PARAMEDIC2 investigators and the original PARAMEDIC2 statisticians (CJ, RL, NS and SG) to determine which adaptive features would be practically feasible to incorporate into the Bayesian designs and how the stopping criteria should be constructed. The statistician remained blind to the trial results until the Bayesian designs’ operating characteristics had been obtained.

#### Interim analysis schedule

The interim analysis schedules explored for the Bayesian designs (B1, B2, B3) are given in Table 1. The maximum sample size was chosen to be the same as the original planned sample size for PARAMEDIC2 (*N* = 8000).

**Table 1:** Bayesian group sequential designs explored for the PARAMEDIC2 study

Interim analysis (*i*)	Stopping boundaries		Number of patients recruited		
	adrenaline better	placebo better	B1 interim analysis schedule^a^	B2 interim analysis schedule	B3 interim analysis schedule
1	0.9999	0.99999	50	50	500
2	0.9998	0.99999	400	300	1000
3	0.9997	0.99998	1100	600	1500
4	0.9996	0.9998	1800	1000	2000
5	0.9995	0.9997	2400	1450	2500
6	0.9994	0.9996	3100	1900	3000
7	0.9993	0.9995	3800	2650	3500
8	0.9992	0.9994	4500	3650	4000
9	0.9991	0.9993	5200	5000	4500
10	0.999	0.9992	5900	6500	5000
11	0.998	0.999	NA	7000	5500
12	0.996	0.998	NA	7500	6000
13	0.994	0.997	NA	NA	6500
14	0.992	0.996	NA	NA	7000
15	0.99	0.994	NA	NA	7500
Final analysis^b^	0.977	0.977	Max 8000	Max 8000	Max 8000

^a^Approximately where the interim analyses occurred since these were on a time basis (3-monthly) and the recruitment was simulated using a Poisson process

^b^If the trial did not stop early, then the final analysis was performed once 8000 patients had been recruited and followed up; if the trial stopped early, then the final analysis was performed once the recruited patients had been followed up

Initially we used the same interim analysis schedule as the original trial design, in terms of the frequency of analyses. That is, a maximum of 10 interim analyses performed 3-monthly, beginning at 50 patients recruited (Design B1, Table 1). When simulating the designs’ operating characteristics, a Poisson process was used to simulate patient recruitment (see below for more details) and so the number of patients at each interim analysis will differ slightly between simulated trial runs.

We then explored the operating characteristics and preference of clinicians of a number of different interim analysis schedules before settling on two designs (B2 and B3) that had their interim analyses based on the number of patients recruited, rather than on a time basis (Design B1). For Design B2, we used the predicted number of patients from the original design (Additional file 1: Table A1.1) for interim analyses 1–10 and then added two additional interim analyses at 7000 and 7500 patients. Design B3 had interim analyses every 500 patients. Both Designs B2 and B3 had an increased number of interim analyses compared to Design B1.

The interim analyses performed in the Bayesian designs involved estimating the posterior distribution for the 30-day survival rates for each arm. Similar to the original design, our Bayesian group sequential designs assumed that stopping early was driven by the primary outcome alone, and other safety outcomes were not considered here. The stopping decisions were based on the posterior probability of superiority (adrenaline better) and harm (placebo better). Further explanation on the stopping rules is provided below.

#### Simulation settings

Simulations of the Bayesian designs were performed in FACTS (version 6.2 [14];) so that their operating characteristics could be studied. Uncertainty at the design stage existed regarding the effect size and survival rates, so we simulated a range of different effect size scenarios for each design. Based on published data available at the time of the design of PARAMEDIC2 (*see* Additional file 2), we assumed survival rates of 2%, 3% and 6%, and simulated scenarios with no improvement (“null”), a 1% improvement and a 2% improvement from each of these 30-day survival rates. Superiority of each arm from each survival rate was simulated separately. The scenarios simulated are summarised in Table 2.

**Table 2:** Scenarios explored for designs when simulating operating characteristics

	Placebo survival rate	Adrenaline survival rate
30-day survival rate 6%	6%	6%
	7%	6%
	6%	7%
	8%	6%
	6%	8%
30-day survival rate 3%	3%	3%
	4%	3%
	3%	4%
	5%	3%
	3%	5%
30-day survival rate 2%	2%	2%
	3%	2%
	2%	3%
	4%	2%
	2%	4%

We assumed a mean recruitment rate of 53 patients/week, which was the predicted average from the trial protocol. We assumed reaching the maximum recruitment rate would take 6 months. Similar to the original design, we assumed no dropouts. Recruitment was simulated stochastically in FACTS using a Poisson process that incorporates the above-mentioned recruitment parameters. We also explored the effect of faster (average 80 patients/week; 1.5 times faster) and slower (average 25 patients/week; half as fast) recruitment rates on the operating characteristics for Bayesian Design B1 since its interim analyses occurred on a time basis (*see* Additional file 3). A benefit of our approach is that we have allowed for uncertainty in the recruitment rate.

Patients who had not completed the 30-day follow-up at each interim analysis had their responses imputed from the posterior distribution. We allowed for primary outcome follow-up to be completed if the trial stopped recruitment early and a final analysis was performed once recruited patients had been followed up for the 30-day post-randomisation period (overrunning).

The type I error was estimated using the proportion of simulations that incorrectly declared a difference between the arms when no difference was present in the true primary outcome rates. We simulated 10,000 trials for the scenarios of no effect to accurately estimate the type I error, and 1000 trials for the other effect sizes to reduce computational burden. The power/probability of declaring a difference was calculated as the proportion of simulations that declared the correct arm to be superior, when one treatment was superior in the true primary outcome rate.

An important operating characteristic is the probability of a "flip-flop". This occurs when the trial stops early due to crossing a stopping boundary at an interim analysis with some of the recruited patients having incomplete primary outcome data, but once the enrolled patients are followed up to 30 days post-randomisation and the final analysis is performed, the critical value specified for declaring a difference at the final analysis is not met. This critical value at the final analysis may be a different value to the stopping boundary that was used at the final interim analysis. Since we were allowing for follow up of patients who had not completed the primary outcome follow-up period at the interim analysis that crossed the stopping boundary (“overrunning analysis”), we wanted to ensure that the probability of having a “flip-flop” was small (< 0.5%) in the Bayesian designs. This was achieved via the choice of stopping boundaries.

#### Prior distributions

One of the features of a Bayesian approach is the ability to formally incorporate information from previous studies and/or the opinions of clinicians. A number of sources were available from which we could construct informative priors and compare the influence of these priors on the designs’ operating characteristics.

In FACTS, normal distributions were used for the priors for the log-odds of the 30-day survival rate for each arm. Initially we used a prior that had a mean 30-day survival rate of 7% and a variance which produced a 95% credible interval of 2–15% on the 30-day survival rate. Identical independent priors were used for both arms, and so we did not assume either arm was superior in the prior distributions. This prior was equivalent to approximately 65 patients’ worth of information in each arm. The decision boundaries described above were chosen on the basis of their operating characteristics using this prior distribution; different decision boundaries would have been chosen under more informative priors.

We then explored the effect of incorporating information from previous studies that was available at the time of the original design for PARAMEDIC2, as well as the opinions of the PARAMEDIC2 clinicians, into the analysis via the prior distributions. The full details and results are given in Additional file 2. Some differences exist in the type I errors, probability of declaring a difference between arms and the expected sample sizes across the priors, and therefore, care should be taken when choosing the stopping boundaries and prior distributions to be used for the design. Freedman and Spiegelhalter [15] demonstrated the influence that the choice of prior (in conjunction with the planned sample size) had on Bayesian stopping boundaries to demonstrate control of type I error.

#### Decision criteria

At each interim analysis, the trial could stop on grounds of efficacy if the posterior probability that the adrenaline arm was superior was greater than its efficacy stopping boundary. The trial could also stop for adrenaline being harmful if the posterior probability that the placebo arm was superior was greater than its stopping boundary. If neither stopping boundary was met, then the trial continued recruiting. The stopping boundaries are given in Table 1. The same boundaries were used at each interim analysis number, but these analyses occurred at a different number of patients recruited across the designs, and Designs B1 and B2 did not use all 15 of the stopping boundary values given in Table 1. For example, interim analysis 3 was performed at approximately 1100, 600, and 1500 patients recruited in Designs B1, B2 and B3, respectively. One could instead choose the stopping boundaries so that similar values are used across the designs based on the number of patients recruited for that analysis. We chose to alter the stopping boundaries based on the interim analysis number, rather than the number of patients recruited, so that we could compare the interim analysis schedules across the designs for the same thresholds. At the final analysis, once follow-up of all recruited patients was complete, a difference between the two arms was declared if the posterior probability that either arm was superior was above 0.977 (*see* Table 1).

Similar to the original trial, we used asymmetric stopping boundaries with stricter values for stopping early for adrenaline being harmful (placebo superior). We began by using stopping boundary values that were equal to 1 minus the nominal *p*-values that were originally proposed for the frequentist PARAMEDIC2 design (Additional file 1: Table A1.1) in our trial simulations. We then explored the effect that increasing and decreasing these stopping boundaries had on the proportion of simulations that stopped early for efficacy or harm, and the type I error and power.

The values in Table 1 were chosen based on the results of simulated trials to produce a two-sided type I error of approximately 5% for each design under a range of assumed 30-day survival rates and > 90% power for the target treatment effect (6% vs 8% 30-day survival rates). Different stopping boundaries could potentially be used to give similar operating characteristics.

One of the main operating characteristics for consideration in our Bayesian designs was the potential for “flip-flops” to occur (defined above). The Bayesian designs were constructed to ensure that there was a low probability of this occurring via the choice of stopping boundaries used.

#### Virtual re-execution of PARAMEDIC2

The PARAMEDIC2 trial was virtually re-executed by reading the trial data into FACTS and applying the Bayesian group sequential designs. At each interim analysis, accumulated trial data were analysed to determine whether the trial should be stopped early.

In the execution of Bayesian design B1, we used the same data that was used in the actual trial interim analyses, since these were performed at the same (calendar) times. In the virtual executions of Designs B2 and B3, we assumed that it took 14 days to collect the data for the primary outcome and have it available for analysis. For interim analyses conducted less than 44 (30 + 14) days after a patient’s recruitment date, it was assumed that the patient’s primary outcome was unknown at that analysis.

## Results

### Operating characteristics of Bayesian designs

**Fig. 1:**
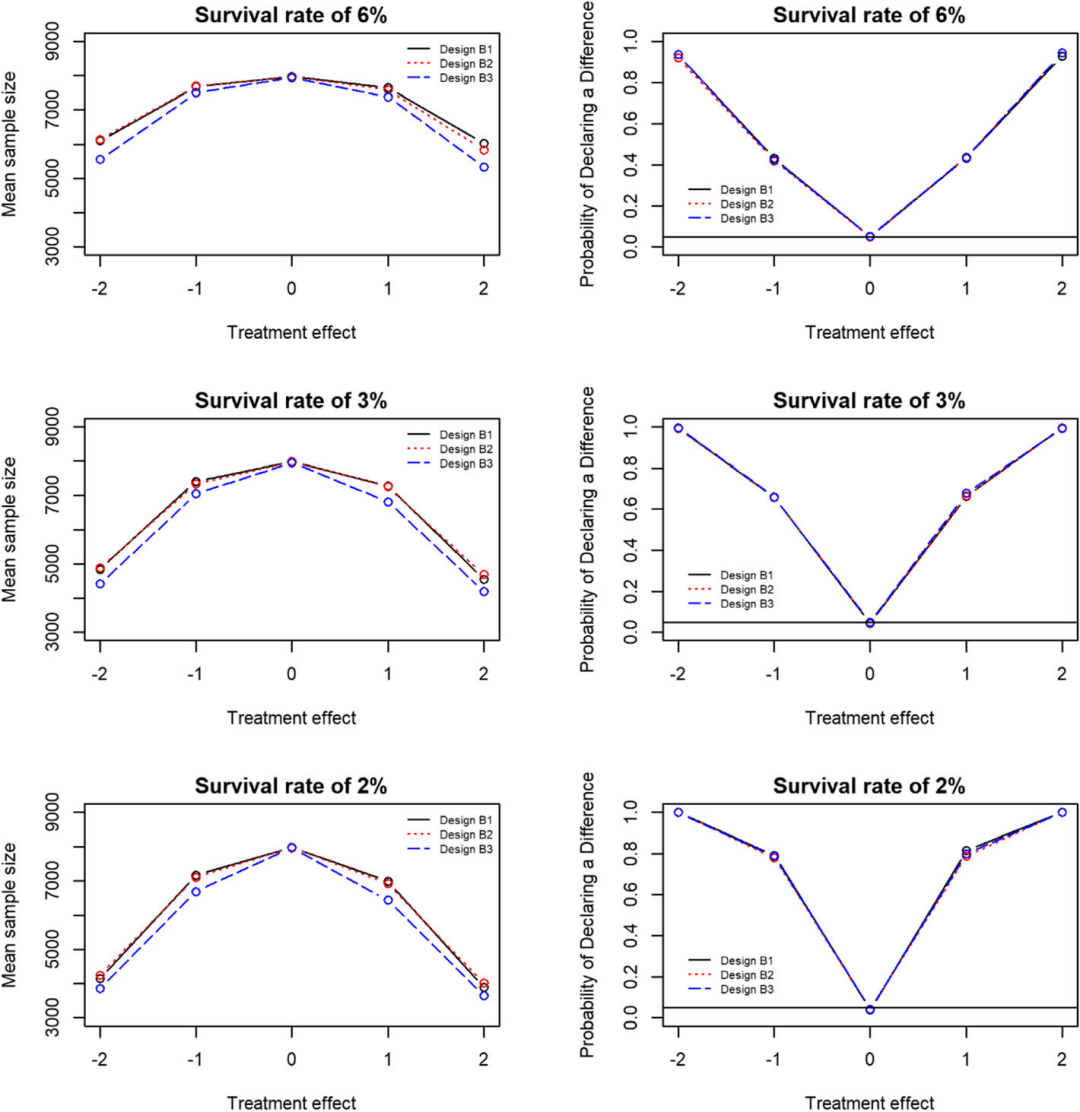
Key operating characteristics for Bayesian designs across several treatment effects and survival rates. The mean sample size is shown in the left column and the probability of declaring a difference between the trial arms is shown in the right column. The treatment effect is the raw difference between adrenaline and placebo survival rates. A positive treatment effect corresponds to adrenaline being superior; a negative treatment effect corresponds to adrenaline being harmful. The horizontal line in the right column figures represents a type I error of 5%

Expected sample sizes and operating characteristics for designs B1, B2 and B3, using the prior introduced above in each arm, are given in Table 3 and Fig. 1. Bayesian design B3 (interim analyses every 500 patients) had the lowest expected sample size for each effect size. Bayesian designs B1 and B2 had similar expected sample sizes (*see* Fig. 1 and Table 3). The type I error increased with the assumed 30-day survival rate, and therefore, these designs may not be controlled at the 5% level if, say, both arms had 8% (or higher) survival rates. Further simulations of the designs would be required if one wished to control type I error over a wider range of assumed survival rates, and different stopping boundaries or sample sizes may be required to give control of the type I error rate and power.

**Table 3:** Operating characteristics for Bayesian group sequential designs for PARAMEDIC2

Design and scenarios^a^	Average duration (weeks)	Average sample size (sd)	Proportion stopped early^b^	Overall proportion declaring a difference^c^	Proportion that did not declare a difference	Proportion flip-flop ^d^	Average probability adrenaline superior
Bayesian Design 1 (B1)							
Null: Placebo 6% vs Adrenaline 6%	168	7968 (390)	0.0074	*0.0493*	0.9506	0.0001	0.5004
Placebo 8% vs Adrenaline 6%	133	6100 (2075)	0.502	0.935	0.064	0.001	0.0072
Placebo 6% vs Adrenaline 8%	131	6019 (2107)	0.525	0.928	0.072	0	0.9932
Placebo 7% vs Adrenaline 6%	163	7676 (1149)	0.084	0.431	0.569	0	0.0995
Placebo 6% vs Adrenaline 7%	162	7654 (1174)	0.09	0.433	0.567	0	0.8961
Null: Placebo 3% vs Adrenaline 3%	168	7980 (293)	0.0053	*0.044*	0.956	0	0.5039
Placebo 5% vs Adrenaline 3%	109	4842 (1985)	0.779	0.994	0.006	0	0.0009
Placebo 3% vs Adrenaline 5%	103	4546 (1960)	0.828	0.995	0.004	0.001	0.9991
Placebo 4% vs Adrenaline 3%	158	7410 (1396)	0.17	0.659	0.341	0	0.0435
Placebo 3% vs Adrenaline 4%	155	7285 (1524)	0.202	0.663	0.337	0	0.9571
Null: Placebo 2% vs Adrenaline 2%	168	7987 (227)	0.004	*0.0371*	0.9629	0	0.5028
Placebo 4% vs Adrenaline 2%	96	4140 (1641)	0.908	0.999	0.001	0	0.0004
Placebo 2% vs Adrenaline 4%	91	3883 (1589)	0.934	1	0	0	0.9995
Placebo 3% vs Adrenaline 2%	153	7172 (1587)	0.234	0.792	0.208	0	0.0238
Placebo 2% vs Adrenaline 3%	150	6991 (1709)	0.288	0.814	0.186	0	0.9785
Bayesian Design 2 (B2)							
Null: Placebo 6% vs Adrenaline 6%	168	7961 (440)	0.0121	*0.0484*	0.9516	0	0.5001
Placebo 8% vs Adrenaline 6%	134	6137 (1911)	0.706	0.92	0.08	0	0.0085
Placebo 6% vs Adrenaline 8%	128	5836 (1984)	0.79	0.943	0.057	0	0.9936
Placebo 7% vs Adrenaline 6%	163	7695 (978)	0.144	0.419	0.581	0	0.1132
Placebo 6% vs Adrenaline 7%	161	7584 (1217)	0.158	0.436	0.564	0	0.9039
Null: Placebo 3% vs Adrenaline 3%	168	7980 (296)	0.0075	*0.0467*	0.9533	0	0.5006
Placebo 5% vs Adrenaline 3%	110	4882 (1932)	0.901	0.991	0.008	0.001	0.0011
Placebo 3% vs Adrenaline 5%	106	4689 (1903)	0.938	0.996	0.004	0	0.9991
Placebo 4% vs Adrenaline 3%	156	7343 (1375)	0.282	0.658	0.341	0.001	0.0524
Placebo 3% vs Adrenaline 4%	155	7260 (1430)	0.313	0.665	0.335	0	0.9587
Null: Placebo 2% vs Adrenaline 2%	168	7984 (242)	0.0079	*0.0411*	0.9589	0	0.5014
Placebo 4% vs Adrenaline 2%	98	4248 (1706)	0.974	1	0	0	0.0004
Placebo 2% vs Adrenaline 4%	94	4019 (1674)	0.983	1	0	0	0.9996
Placebo 3% vs Adrenaline 2%	152	7106 (1460)	0.406	0.779	0.221	0	0.0257
Placebo 2% vs Adrenaline 3%	148	6928 (1621)	0.443	0.789	0.211	0	0.978
Bayesian Design 3 (B3)							
Null: Placebo 6% vs Adrenaline 6%	167	7936 (492)	0.027	*0.0515*	0.9481	0.0004	0.5003
Placebo 8% vs Adrenaline 6%	123	5562 (1879)	0.827	0.935	0.065	0	0.0066
Placebo 6% vs Adrenaline 8%	118	5333 (1829)	0.87	0.945	0.055	0	0.9924
Placebo 7% vs Adrenaline 6%	159	7497 (1183)	0.235	0.424	0.574	0.002	0.0959
Placebo 6% vs Adrenaline 7%	157	7382 (1216)	0.303	0.435	0.562	0.003	0.9017
Null: Placebo 3% vs Adrenaline 3%	168	7957 (293)	0.0223	*0.0492*	0.9505	0.0003	0.5002
Placebo 5% vs Adrenaline 3%	101	4416 (1587)	0.976	0.995	0.005	0	0.011
Placebo 3% vs Adrenaline 5%	97	4186 (1535)	0.982	0.993	0.006	0.001	0.9985
Placebo 4% vs Adrenaline 3%	151	7052 (1475)	0.431	0.66	0.338	0.002	0.043
Placebo 3% vs Adrenaline 4%	146	6821 (1512)	0.53	0.678	0.32	0.002	0.9552
Null: Placebo 2% vs Adrenaline 2%	168	7970 (282)	0.0173	*0.0415*	0.9576	0.0009	0.4979
Placebo 4% vs Adrenaline 2%	90	3854 (1364)	0.996	0.999	0.001	0	0.0006
Placebo 2% vs Adrenaline 4%	87	3643 (1327)	0.996	0.999	0.001	0	0.9993
Placebo 3% vs Adrenaline 2%	144	6691 (1594)	0.564	0.788	0.21	0.002	0.0217
Placebo 2% vs Adrenaline 3%	139	6436 (1592)	0.66	0.80	0.198	0.002	0.9776

^a^Different effect size scenarios that were simulated for each design are given as placebo 30-day survival rate vs adrenaline 30-day survival rate

^b^Proportion of simulations that stopped early and were declared to have a difference (in the correct direction) at the final analysis

^c^The simulated type I errors are italicised

^d^These simulations were stopped early for efficacy or harm, but they did not meet the critical values to declare a difference between the treatments at the final analysis once all patients were followed up (insufficient evidence of a difference)

Each of the Bayesian designs had greater than 90% probability to declare the correct arm to be superior when there was a treatment effect of a 2% difference (when either arm was superior). They had a low probability (42–44%) to detect an improvement from 6% to 7% (RR 1.17) 30-day survival, and a slightly higher probability (66–68%) to detect an improvement from 3% to 4% 30-day survival (RR 1.33). The Bayesian designs had approximately 80% probability to detect an improvement from 2% to 3% 30-day survival (RR 1.5) and declare the correct arm to be superior.

Initially, when we used 1 – nominal *p*-values that were originally proposed for the frequentist design (given in Additional file 1: Table A1.1) for the stopping boundaries for the Bayesian designs, we found there to be a high proportion of flip-flops (up to 9%; *see* Additional file 4). We therefore used strict stopping boundaries (Table 1), which reduced the chance of early stopping and the proportion of flip-flops. This led to higher average sample sizes but also gave higher power. In a frequentist trial design the critical value for the final analysis would be updated for trials that stopped early to account for the unspent alpha and observed information, and so for this trial, the proportion of flip-flops for a frequentist design might not be as high as the Bayesian version of the frequentist design would suggest. Example single trial simulations for each design are provided in Additional file 5.

### Re-executing PARAMEDIC2 with Bayesian group sequential designs

**Fig. 2:**
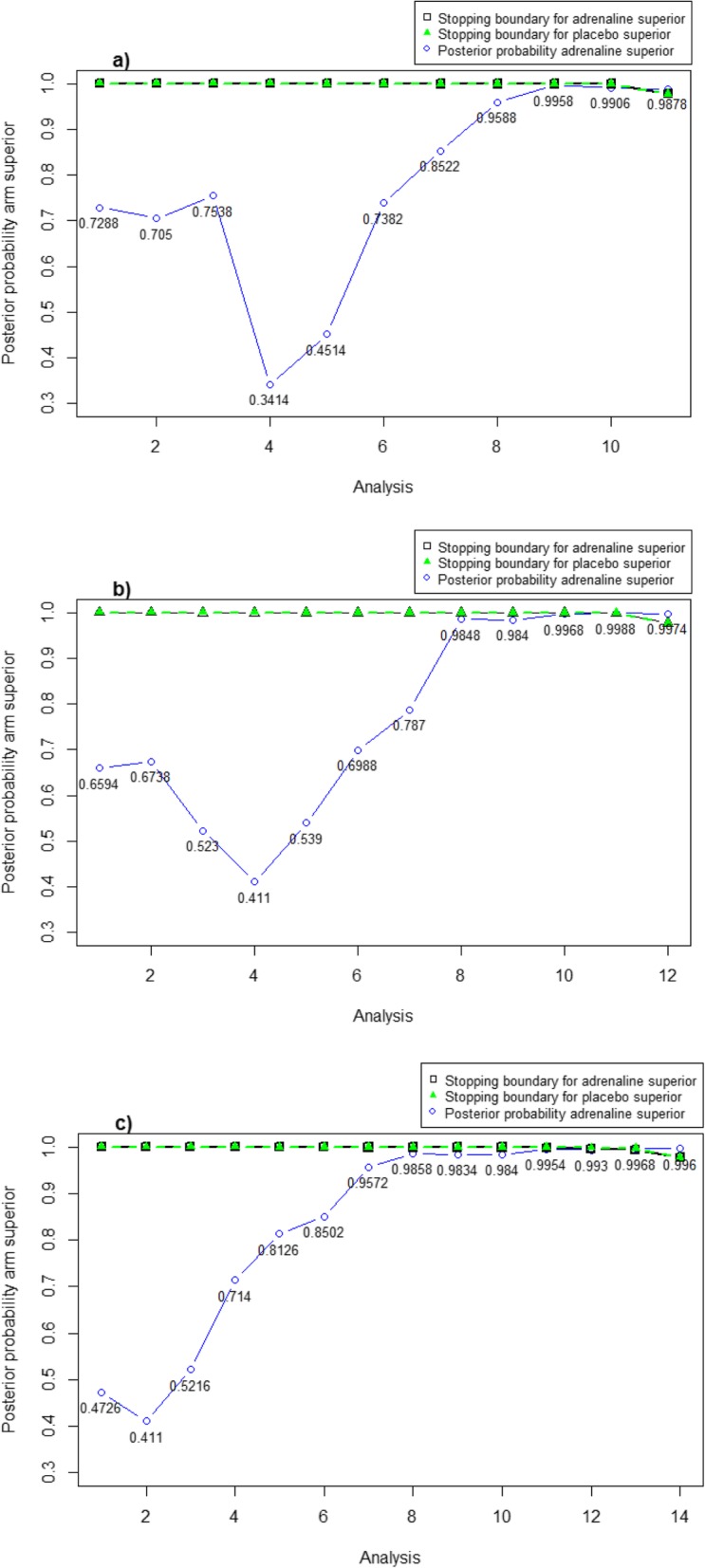
Virtual execution of Bayesian designs B1-B3 using the PARAMEDIC2 data. **a**) Bayesian design B1, **b**) Bayesian design B2; **c**) Bayesian design B3. The posterior probability of having adrenaline superior at each analysis is displayed as an open circle. The posterior probability of having placebo superior is 1 – the probability adrenaline is superior. The stopping boundaries for adrenaline superior are given as the black solid line with open squares; the stopping boundaries for placebo superior/adrenaline harmful are given as the green dotted line with closed triangles. The x-axis displays the analysis number and so the number of patients at each analysis generally differs between the designs

The results of the interim analyses from the virtual executions for each design are presented in Fig. 2 and Additional file 6: Tables A6.1–6.3. The virtual executions used the same prior that was used to generate the operating characteristics in Table 3. A prior sensitivity analysis was conducted during the virtual executions of the Bayesian designs, and the results are presented in Additional file 6: Tables A6.4-S6.6.

When implementing Bayesian design B1, none of the interim analyses recommended stopping the trial early (when the above-mentioned prior was used). Very few survival events occurred in the earlier interim analyses, and these did not provide much information; the posterior estimates were closer to the prior mean at these interim analyses. The posterior probabilities that adrenaline was superior came close to the stopping boundaries in the later interim analyses for Bayesian design B1, and at the final analysis, the trial crossed the decision threshold with a posterior probability of 0.9878 that adrenaline was superior.

Design B2 recommended stopping early for declaring adrenaline superior at interim analysis 11 at 7000 patients, and Design B3 recommended stopping early for declaring adrenaline superior at interim analysis 13 at 6500 patients. The inferences resulting from the designs that resulted in decreased sample sizes are similar to those of the original trial. For Bayesian designs B2 and B3, there were posterior probabilities of 0.9974 and 0.996, respectively, that adrenaline was superior at the final analysis.

## Discussion

Through choice of the stopping boundaries, the Bayesian group sequential designs we proposed had greater than 90% power for the target treatment effect, a low probability of having “flip-flops,” and approximately 5% type I error. The Bayesian design that had fixed sample size increments of 500 patients (Design B3) tended to produce the lowest average sample size of the three Bayesian designs investigated. This design presents a trade-off in the potential for a lower average sample size at the cost of increased operational complexity due to a higher number of interim analyses. A disadvantage of this design is that it has the latest first interim analysis, which would be problematic if one of the arms was causing harm. Each of the Bayesian designs had similar probabilities of declaring a difference between the arms for each of the effect sizes studied.

When virtually re-executing PARAMEDIC2 using the Bayesian designs and the trial data, we found that if more interim analyses were taken later during recruitment, the PARAMEDIC2 trial could have stopped early, declaring adrenaline superior with approximately 1500 fewer patients. It appears that the PARAMEDIC2 trial would have benefitted with more interim analyses later on in the trial, once more survival events had been observed. However, the trial recruited approximately 300–500 patients per month after 3000 patients had been recruited, and so data cleaning, analysis and planning the Data Monitoring Committee (DMC) meetings may have been practically difficult for Designs B2 and B3, which had more interim analyses later on in recruitment.

The simulation approach implemented in this paper is very flexible because it enables one to explore the operating characteristics of different design options (interim analysis schedules, stopping boundary values, decision criteria) under various possible scenarios (true effect size, variability of the primary outcome, control arm rate, recruitment rate). The design to be implemented can then be chosen based on the operating characteristics it produces under a range of scenarios. Simulation of trial designs is important in both the Bayesian and frequentist frameworks, but since more “off the shelf” frequentist group sequential designs are available, simulation is not as routinely performed as when constructing Bayesian designs. Approaches such as sample size re-estimation could also be used as an alternative to information-based group sequential designs in scenarios where there is uncertainty in event rates.

The Bayesian approach allowed us to incorporate the opinions of clinicians and information from previous studies on the effect size via the prior distributions. It also enabled us to use decision criteria that were based on the probability of benefit or harm, which are more clinically relevant than *p*-values. Additional benefits may be gained by using a Bayesian approach for more complex designs, such as multi-arm trials that use response adaptive randomisation or those with longitudinal or multi-level modelling since they can incorporate multiple complex decisions [16]. A recent example is the REMAP-CAP trial [17] which is a Bayesian adaptive platform trial for patients with community-acquired pneumonia that is currently recruiting.

The designs presented in this paper are situation-specific, as all adaptive designs are, and if different clinicians had been consulted, different designs would have been investigated. We do not recommend simply taking the stopping boundaries from Table 1 and using them in other trials without first studying the operating characteristics of the designs in different trial contexts. We chose stopping boundary values to produce a simulated type I error of approximately 5% and a low proportion of flip-flops. However, not all Bayesians are concerned with the control of type I error as this is a frequentist property. Also, not all designs may allow for overrunning analyses (i.e. do not collect follow-up data on incomplete patients once the trial has stopped early). If less stringent values had been used for the stopping boundaries, smaller expected sample sizes would have been obtained in the trial simulations and different decisions are likely to have been made at the interim analyses when virtually executing the trials.

Our Bayesian designs assumed that stopping early for the superiority of adrenaline or for adrenaline being harmful was driven by the primary outcome. We had considered also using a secondary outcome from the trial, the modified Rankin Scale (mRS), which measures neurological and cognitive outcomes, in the decision-making process. Given the low survival rate for PARAMEDIC2 the mRS did not provide much more information at the interim analyses than the survival outcome. Quantification of a desirable effect size was also difficult since there was little known about the distribution of the mRS for OHCA patients. Similar to the original trial, the DMC could examine additional safety data and make deviations to ensure patient safety if required.

The software that was used to simulate the designs’ operating characteristics and perform the virtual executions of the trial (FACTs) is a commercial software that is only one of a number of possible options. Grayling and Wheeler [18] provide a review of available software for adaptive clinical trial designs.

## Conclusions

We have demonstrated how a Bayesian group sequential approach could be used to design a phase III emergency medicine trial. We also demonstrated that for this case study, later interim analyses would most likely have led to early stopping to declare adrenaline superior for 30-day survival with a high probability, thus reducing the sample size of the PARAMEDIC2 study.

## Supplementary information

**Additional file 1:** Original trial design and Interim analysis results from PARAMEDIC2 trial.

**Additional file 2:** Incorporating prior information.

**Additional file 3:** Effect of varying recruitment rate on the operating characteristics of Design B1.

**Additional file 4:** Operating characteristics for Bayesian design based on frequentist stopping boundaries.

**Additional file 5:** Example trials.

**Additional file 6:** Re-executing PARAMEDIC2 with Bayesian group sequential designs.

## Data Availability

The data used in this study were generated as part of the PARAMEDIC2 study. Requests to share individual, de-identified participant data, aggregated data, data dictionaries and other study documents from this study should be sent to the PARAMEDIC2 CI (Gavin Perkins; paramedictrial@warwick.ac.uk). The data are archived at Warwick University and will likely be available whilst the data guardians (R Lall, C Ji and G Perkins) are employed by this institution. Data-sharing requests will be assessed on their individual merits by the Steering Committee and compliance with the University of Warwick’s Standard Operating Procedures on Data Management and Sharing. Other documents relating to this secondary analysis may be available on request from the lead researcher (EG Ryan). Requests for documents will be assessed on their individual merits.

## Abbreviations

DMC: Data Monitoring Committee
FACTS: Fixed and Adaptive Clinical Trial Simulator
FDA: Food and Drug Administration
mRS: modified Rankin Scale
OHCA: out-of-hospital cardiac arrest
PARAMEDIC2: The Prehospital Assessment of the Role of Adrenaline: Measuring the Effectiveness of Drug administration In Cardiac arrest study
RCT: randomised controlled trial
REBOA: Resuscitative endovascular balloon occlusion of the aorta
RR: relative risk
