# Supplementary material for: Bayesian group sequential designs for phase III emergency medicine trials: a case study using the PARAMEDIC2 trial
Source: Trials. 2020 Jan 14;21:84. doi: 10.1186/s13063-019-4024-x (PMC6961266; doi:10.1186/s13063-019-4024-x)
Supplement: Supplementary file 1 — Additional file 1: Original trial design and Interim analysis results from PARAMEDIC2 trial. [file 13063_2019_4024_MOESM1_ESM.pdf]

Additional File 1 - Original trial design and Interim analysis results from PARAMEDIC2 trial

Table A1.1 shows the predicted total number of patients that would be recruited at each interim analysis. Most of the interim analyses were projected to occur early in the trial, in terms of the number of patients recruited. These were calculated assuming 30-day survival of 6% and 8% in the placebo and adrenaline arms, respectively.

Table A1.1. P-values corresponding to the stopping boundaries that were originally proposed for the PARAMEDIC2 trial for analyses to be performed at 3 monthly intervals and the final analysis

Interim analysis	Predicted total number patients recruited (information fraction)	p-value for upper bound (adrenaline superior)	p-value for lower bound (placebo superior/adrenaline harmful)
**1**	50 (0.006)	0.0036	<0.00001
**2**	300 (0.038)	0.0040	<0.00001
**3**	600 (0.075)	0.0044	0.00002
**4**	1000 (0.125)	0.0048	0.0002
**5**	1450 (0.181)	0.0052	0.0008
**6**	1900 (0.238)	0.0055	0.0021
**7**	2650 (0.331)	0.0057	0.0042
**8**	3650 (0.456)	0.0060	0.0069
**9**	5000 (0.625)	0.0062	0.0103
**10**	6500 (0.813)	0.0064	0.0142
**Final analysis**	8000 (1.00)	0.0065	0.0184

The PARAMEDIC2 study had slower recruitment and lower survival rates than originally anticipated. The stopping boundaries and critical p-values depend on the assumed information (e.g., number of patients recruited, number of events) and so they were updated during the trial to reflect the amount of information at each interim analysis using the pre-specified alpha-spending functions. The p-values associated with the updated chi-square boundaries are given in columns 3-4 of Table A1.2. Column 5 provides the calculated p-values for the trial.

Table A1.2 Interim analysis results for original PARAMEDIC2 trial

Interim analysis	Number of patients recruited (placebo:adrenaline)	Adjusted P-value for upper bound (adrenaline superior) for obtained information	Adjusted P-value for lower bound (placebo superior) for obtained information	Calculated P-value for analysis
**1**	44 (30:14)	0.00010	<0.00001	0.2511
**2**	97 (58:39)	0.00010	<0.00001	0.3443
**3**	245 (134:111)	0.00012	<0.00001	0.2444
**4**	665 (332:333)	0.00073	<0.00001	0.5459
**5**	1226 (595: 631)	0.00130	<0.00001	0.9180
**6**	2008 (993:1015)	0.00230	<0.00001	0.4259
**7**	2785 (1388:1397)	0.00349	<0.00001	0.2234
**8**	3551 (1757:1794)	0.00481	<0.00001	0.0526
**9**	4737 (2358:2379)	0.00631	<0.00001	0.0085
**10**	6018 (3006:3012)	0.00791	<0.00001	0.0093
**Final analysis**	8014 (3999:4015)	0.025	0.025	0.02
