# Supplementary material for: Bayesian group sequential designs for phase III emergency medicine trials: a case study using the PARAMEDIC2 trial
Source: Trials. 2020 Jan 14;21:84. doi: 10.1186/s13063-019-4024-x (PMC6961266; doi:10.1186/s13063-019-4024-x)
Supplement: Supplementary file 2 — Additional file 2: Incorporating prior information. [file 13063_2019_4024_MOESM2_ESM.pdf]

Additional File 2 - Incorporating prior information

One of the advantages of the Bayesian approach is the ability to formally incorporate information from previous studies and/or the opinions of clinicians. There were a number of sources available from which we could construct informative priors and compare the influence of these different priors on the designs' operating characteristics.

At the time that the PARAMEDIC2 trial was being designed, there was only one previous RCT that compared the use of adrenaline to placebo in out of hospital cardiac arrest patients – the PACA trial [1]. This trial found the survival to hospital discharge to be 4% and 1.9% in the adrenaline and placebo arms, respectively. There were also a number of studies that compared standard dose (1mg) adrenaline (control) to high dose adrenaline or vasopressin (intervention), and so the results from the standard adrenaline dose arm could be used to derive prior distributions for the adrenaline arm in the Bayesian designs (see Table A2.1). A number of observational studies had also been performed which compared the use of adrenaline to not using adrenaline for out of hospital cardiac arrest patients (see Table A2.2). Some of the studies reported survival to discharge rather than 30-day survival, and so it was assumed that these rates were similar.

Table A2.1 Previous RCTs investigating the use of standard dose (1mg) adrenaline to high dose adrenaline or vasopressin

Study	Standard dose adrenaline survival to discharge/30 day survival	Comparator	Setting
Brown et al [2]	26/632 (4.1%)	High dose adrenaline	OHCA
Callaham et al [3]	3/270 (1.1%)	High dose adrenaline	OHCA (non-trauma)
Gueugniaud et al. [4]	46/1650 (2.8%)	High dose adrenaline	OHCA (non-trauma)
Sherman et al. [5]	0/62 (0%)	High dose adrenaline	OHCA (non-trauma), on arrival Emergency department
Steill et al. [6]	2/165 (1.2%)	High dose adrenaline	IHCA/OHCA
Jacobs et al. [1]	11/272 (4.0%)	Placebo	OHCA
Ducros et al. [7]	2/16 (12.5%)	Vasopressin/Epinephrine	Witnessed OCHA
Gueugniaud et al. [8]	33/1452 (2.3%)	Vasopressin / Epinephrine	OHCA (non-trauma)
Lindner et al. [9]	3/20 (15.0%)	Vasopressin / Epinephrine	OHCA, Refractory VF
Ong et al. [10]	8/353 (2.3%)	Vasopressin / Epinephrine	Emergency department
Wenzel et al. [11]	58/597 (9.7%)	Vasopressin / Epinephrine	OHCA (non-trauma)

OHCA = out of hospital cardiac arrest; IHCA = in hospital cardiac arrest; VF = ventricular fibrillation

Table A2.2 Previous observational studies on the use of adrenaline for out of hospital cardiac arrests

Study	Adrenaline survival to discharge/30 day survival	No adrenaline survival to discharge/30 day survival
Hagihara et al. [12]	805/15030 (5.4%)	18906/402158 (4.7%)
Holmberg et al. [13]	156/4566 (3.4%)	388/6207 (6.3%)
Kirves et al. [14]*	29/77 (38%)	60/80 (75%)
Olasveengen et al. [15]	24/367 (7%)	60/481 (13%)
Ong et al. [16]	11/681 (1.6%)	6/615 (1%)
Vayrynen et al. [17]	39/703 (5.5%)	18/86 (20.9%)

Hayashi et al. [18]	137/1013 (13.5%)	258/2148 (12%)
Herlitz et al. [19]	50/417 (12%)	149/786 (19%)

*These survival rates were much higher than the other studies and were not included in the derivation of the prior distributions

We also asked the PARAMEDIC2 investigator clinicians for their opinions on plausible rates of the primary outcome and they believed that a mean 30-day survival rate of 3% should be used, and that a rate above 8% would not be plausible (for either arm).

Since survival at 30 days is a binary variable, we used the Bernoulli distribution to model the primary outcome for each arm:

$$Y_j|t \sim Bernoulli(P_t)$$

where Y_j is the 30-day survival status for patient *j* and P_t is the probability distribution for the 30-day survival rate for arm *t*. Since this is a Bayesian approach, we need prior distributions for P_t.

In FACTS, normal distributions were used for the priors for the log-odds of the 30-day survival rate for each arm *t* (*t*=adrenaline, placebo), $\theta_t = log\left(\frac{P_t}{1-P_t}\right)$. The 30-day survival rate for arm *t*, P_t, is modelled as:

$P_t = \frac{e^{\theta_t}}{1+e^{\theta_t}}, where\ \theta_t \sim N(\mu_t, v_t^2)$, μ_t is the mean rate for arm *t* (on the log-odds scale), and v_t^2 is the variance.

Using the results from previous studies, as well as the opinions of the PARAMEDIC2 clinicians, we were able to derive a number of prior distributions to conduct a prior sensitivity analysis. These are described in Table A2.3.

Table A2.3. Prior distributions for Bayesian group sequential designs for PARAMEDIC2

Prior	Mean 30-day survival rate adrenaline	$\mu_{adrenaline}$, $v_{adrenaline}^2$	Approximate no. observations in adrenaline arm prior	Mean 30-day survival rate placebo	$\mu_{placebo}$, $v_{placebo}^2$	Approximate no. observations in placebo arm prior
**Original prior (P1)**	7%	-2.7, 0.5^2	65	7%	-2.7, 0.5^2	65
**Clinician informed prior (P2)**	3%	-3.5, 0.55^2	107	3%	-3.5, 0.55^2	107

**PACA trial [1] prior (P3)**	4%	-3.2, 0.2^2	660	2%	-3.9, 0.14^2	2580
**Vaguer PACA trial [1] prior (P4)**	4%	-3.2, 0.8^2	35	2%	-3.9, 0.8^2	67
**Previous adrenaline RCTS (placebo worse) (P5)**	3.5%	-3.3, 0.8^2	39	2%	-3.9, 0.8^2	66
**Previous adrenaline RCTS (placebo same) (P6)**	3.5%	-3.3, 0.8^2	39	3.5%	-3.3, 0.8^2	39
**Observational studies (P7)**	7%	-2.8, 0.78^2	27	8%	-2.4, 1.1^2	9

The original prior (P1, Table A2.3), that was used throughout the main manuscript, was centred on the original estimates of 30 day survival at approximately 7%, and had a variance that produced a 95% Credible Interval (CrI) of 2-15% on the 30-day survival rate. The clinician informed prior (P2) had a mean survival rate of 3% and a variance that produced a 95% CrI of 1-8% on the 30-day survival rate.

The results from the PACA trial [1] were used to inform the mean and variance for the "PACA trial prior" (P3). These values were derived from the trial sample proportions and their standard error. Priors were also run with larger variances since the standard errors derived from the PACA trial were quite small – this is the "Vaguer PACA trial prior" (P4).

The weighted mean survival to discharge of the previous RCTs that used standard dose adrenaline as the control (Table A2.1) was 3.5%, and so this was used as the mean for the prior. The variance was chosen to ensure the upper limit of the 95% CrI covered the range of survival rates in the studies. These studies only had information on the adrenaline arm, and so one set of priors was run assuming placebo was worse, with a mean of 2%, and a variance that produced an upper limit of 5% for a 95% CrI on the 30-day survival rate (P5). Another set of priors were run assuming the placebo arm had the same prior as the adrenaline arm (P6).

Priors were derived from the observational studies (Table A2.2) using the mean of the sample proportions across the studies and variance of the proportions for each arm (P7). The Kirves et al. [14] results were not included as these results were very different to the other studies.

Separate simulations were run with each prior listed in Table A2.3 for the designs from Table 1 in the main paper and the results are displayed in Tables A2.4-A2.6 and Figures A2.1-A2.3.

For each Bayesian design, the PACA prior (P3) produced very high simulated type I error rates and

generally had lower power for the “adrenaline harmful” scenarios, compared to the other priors. The PACA prior had much higher power when adrenaline was assumed to have a survival rate 1% higher than placebo, and had similar power to the other priors when adrenaline was assumed to have a survival rate that was 2% higher than placebo. These operating characteristics were produced because this prior was strongly informative and in favour of adrenaline. The average sample size for the PACA prior was also much smaller for the “adrenaline superior” scenarios, and the null scenarios.

The other priors produced similar power to the original prior (P1). The following priors tended to produce smaller average sample sizes than the original prior: clinician prior (P2); priors based on previous RCTs of adrenaline where the placebo arm was assumed to have the same prior as the adrenaline arm (P6); observational studies prior (P7). The vaguer PACA prior (P4) produced smaller average sample sizes for the adrenaline superior scenarios, and similar average sample sizes for the other scenarios compared to the original prior. The priors based on previous adrenaline RCTs where placebo was assumed to perform worse than adrenaline (P5) had higher average sample sizes compared to the original prior for the adrenaline harmful scenarios, and smaller average sample sizes for the adrenaline superior scenarios

For designs B1 and B3, when a survival of 6% was assumed, under the null scenario, priors P2-P7 produced a type I error that was larger than the targeted 5%. When a survival of 3% was assumed for these designs, priors P3-P5 produced a type I error >5% under the null scenario. Only prior P3 produced a type I error >5% under the null scenario when a survival of 2% was assumed for designs B1 and B3.

For design B2, when a survival of 6% or 3% was assumed, under the null scenario, priors P2-P7 produced a type I error that was larger than the targeted 5%. When a survival of 2% was assumed, priors P3-P7 produced a type I error rate that was >5% under the null scenario.

Table A2.4 Prior sensitivity analysis for the Bayesian designs (Design B1)

	Original prior (P1)		Clinician prior (P2)		PACA prior (P3)		PACA prior (vaguer) (P4)		RCT prior (pbo worse) (P5)		RCT prior (pbo same) (P6)		Observational studies (P7)	
	**Average sample size**	**Prop. declaring difference**	**Average sample size**	**Prop. declaring difference**	**Average sample size**	**Prop. declaring difference**	**Average sample size**	**Prop. declaring difference**	**Average sample size**	**Prop. declaring difference**	**Average sample size**	**Prop. declaring difference**	**Average sample size**	**Prop. declaring difference**
**Null: Placebo 6% vs Adrenaline 6%**	7968 (390)	*0.0493*	7960 (454)	0.0511	2988 (3141)	0.7985	7941 (573)	0.0549	7915 (718)	0.0598	7955 (490)	0.0528	7954 (488)	0.0535
**Placebo 8% vs Adrenaline 6%**	6100 (2075)	0.935	6096 (2146)	0.937	5574 (3487)	0.557	6203 (2087)	0.931	6483 (1949)	0.909	6052 (2160)	0.935	5963 (2193)	0.939
**Placebo 6% vs Adrenaline 8%**	6019 (2107)	0.928	5896 (2197)	0.93	814 (686)	1	5659 (2265)	0.938	5151 (2460)	0.954	5929 (2170)	0.932	5981 (2139)	0.928
**Placebo 7% vs Adrenaline 6%**	7676 (1149)	0.431	7667 (1158)	0.426	4492 (3606)	0.504	7667 (1172)	0.415	7752 (997)	0.364	7637 (1192)	0.429	7637 (1214)	0.436
**Placebo 6% vs Adrenaline 7%**	7654 (1174)	0.433	7592 (1265)	0.441	1341 (1601)	0.992	7497 (1437)	0.467	7321 (1669)	0.513	7637 (1205)	0.439	7631 (1212)	0.427
**Null: Placebo 3% vs Adrenaline 3%**	7980 (293)	*0.044*	7973 (365)	0.0477	5696 (3212)	0.4913	7956 (462)	0.0526	7955 (473)	0.0538	7967 (397)	0.0416	7966 (401)	0.0499
**Placebo 5% vs Adrenaline 3%**	4842 (1985)	0.994	4619 (1945)	0.996	7258 (2204)	0.736	4768 (2009)	0.99	5042 (2002)	0.989	4577 (2026)	0.994	4626 (2014)	0.994
**Placebo 3% vs Adrenaline 5%**	4546 (1960)	0.995	4395 (1982)	0.995	1091 (794)	1	4082 (2015)	0.996	3874 (2021)	0.996	4349 (2044)	0.994	4323 (1997)	0.996
**Placebo 4% vs Adrenaline 3%**	7410 (1396)	0.659	7333 (1498)	0.67	6920 (2631)	0.203	7360 (1453)	0.655	7481 (1318)	0.616	7235 (1599)	0.677	7277 (1546)	0.674
**Placebo 3% vs Adrenaline 4%**	7285 (1524)	0.663	7208 (1609)	0.678	2274 (2186)	0.991	6973 (1902)	0.709	6804 (2003)	0.734	6804 (2003)	0.734	7139 (1707)	0.687

**Null: Placebo 2% vs Adrenaline 2%**	7987 (227)	*0.0371*	7978 (301)	0.0434	6806 (2650)	0.2892	7961 (436)	0.0494	7972 (359)	0.0469	7973 (349)	0.0476	7971 (365)	0.0472
**Placebo 4% vs Adrenaline 2%**	4140 (1641)	0.999	3919 (1634)	0.999	7371 (859)	0.905	4022 (1719)	0.999	4157 (1707)	0.999	3817 (1641)	0.999	3842 (1646)	0.999
**Placebo 2% vs Adrenaline 4%**	3883 (1589)	1	3695 (1625)	0.997	1276 (873)	1	3334 (1631)	0.999	3315 (1589)	0.999	3603 (1650)	0.999	3548 (1612)	1
**Placebo 3% vs Adrenaline 2%**	7172 (1587)	0.792	7044 (1679)	0.809	7392 (2069)	0.2	7111 (1665)	0.786	7180 (1603)	0.771	6950 (1779)	0.815	7031 (1734)	0.809
**Placebo 2% vs Adrenaline 3%**	6991 (1709)	0.814	6836 (1839)	0.822	2921 (2396)	0.99	6499 (2102)	0.852	6502 (2045)	0.85	6757 (1910)	0.827	6684 (1948)	0.836

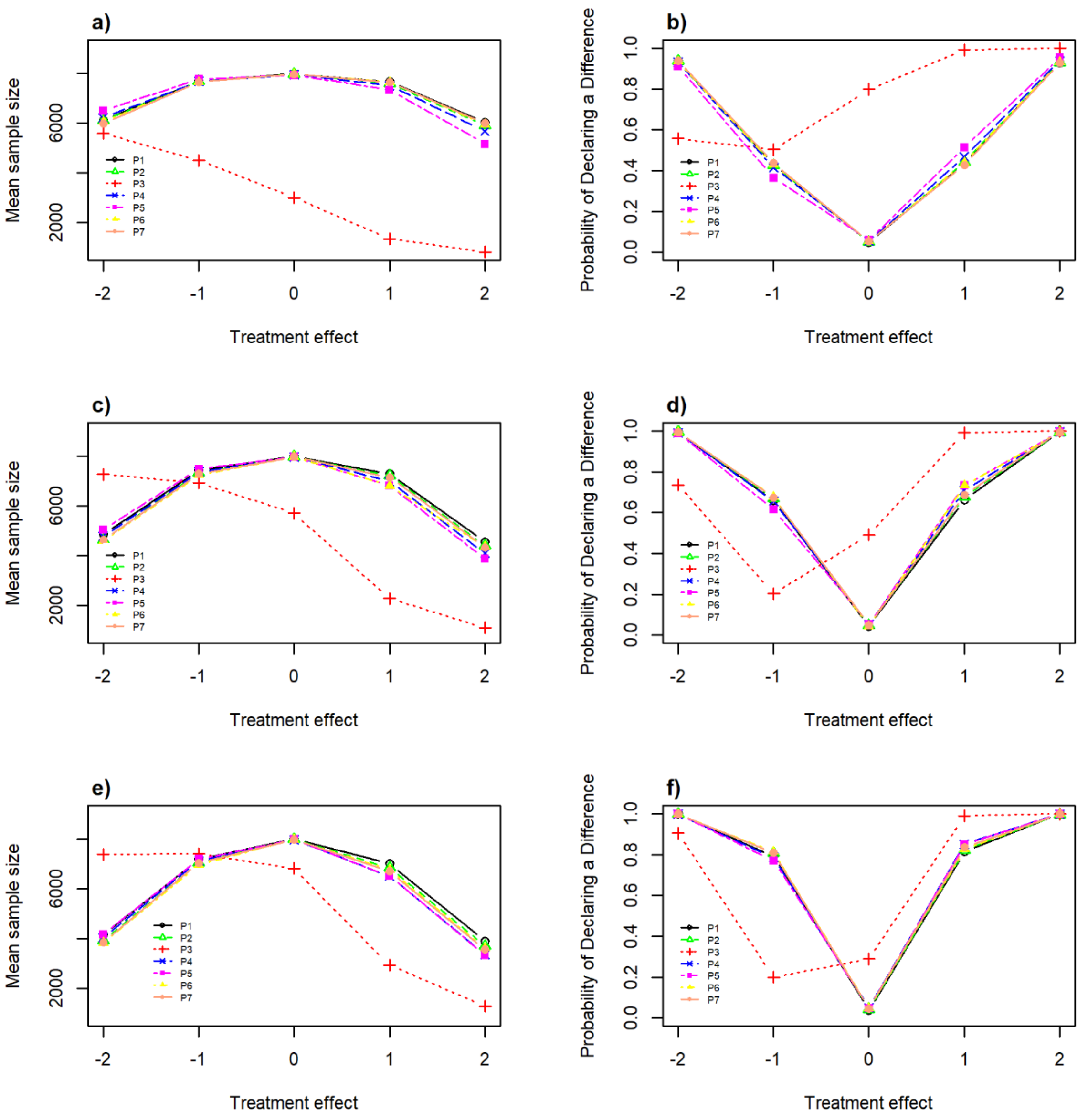

Figure A2.1. Mean sample size (left column) and probability of declaring a difference between arms (right column) for Design B1 for each prior (P1-P7) across a range of treatment effects (difference between adrenaline and placebo survival rates) and different assumed survival rates. A positive treatment effect corresponds to adrenaline being superior; a negative treatment effect corresponds to adrenaline being harmful. Figures a) and b) correspond to a control survival rate of 6%; figures c) and d) correspond to a control survival rate of 3%; figures e) and f) correspond to a control survival rate of 2%.

Table A2.5 Prior sensitivity analysis for the Bayesian designs (Design B2)

	Original prior (P1)		Clinician prior (P2)		PACA prior (P3)		PACA prior (vaguer) (P4)		RCT prior (pbo worse) (P5)		RCT prior (pbo same) (P6)		Observational studies (P7)	
	Average sample size	Prop. declaring difference	Average sample size	Prop. declaring difference	Average sample size	Prop. declaring difference	Average sample size	Prop. declaring difference	Average sample size	Prop. declaring difference	Average sample size	Prop. declaring difference	Average sample size	Prop. declaring difference
**Null: Placebo 6% vs Adrenaline 6%**	7961 (440)	*0.0484*	7947 (522)	0.0516	2500 (3033)	0.8296	7918 (692)	0.0543	7865 (916)	0.0644	7938 (587)	0.0525	7946 (540)	0.0504
**Placebo 8% vs Adrenaline 6%**	6137 (1911)	0.92	6045 (1981)	0.919	4934 (3660)	0.575	6226 (1909)	0.914	6508 (1729)	0.894	6044 (2008)	0.921	5975 (2046)	0.923
**Placebo 6% vs Adrenaline 8%**	5836 (1984)	0.943	5703 (2072)	0.941	620 (494)	1	5491 (2209)	0.942	4903 (2416)	0.96	5641 (2138)	0.942	5776 (2031)	0.941
**Placebo 7% vs Adrenaline 6%**	7695 (978)	0.419	7645 (1093)	0.428	3761 (3611)	0.603	7699 (997)	0.404	7753 (972)	0.36	7653 (1073)	0.427	7670 (1050)	0.424
**Placebo 6% vs Adrenaline 7%**	7584 (1217)	0.436	7514 (1255)	0.44	995 (1328)	0.996	7402 (1459)	0.461	7124 (1881)	0.519	7518 (1267)	0.444	7563 (1177)	0.427
**Null: Placebo 3% vs Adrenaline 3%**	7980 (296)	*0.0467*	7961 (426)	0.0519	5197 (3422)	0.533	7941 (554)	0.0556	7944 (523)	0.0539	7948 (513)	0.0549	7956 (459)	0.054
**Placebo 5% vs Adrenaline 3%**	4882 (1932)	0.991	4725 (1979)	0.994	6768 (2569)	0.748	4846 (1983)	0.994	5115 (1924)	0.987	4646 (2021)	0.998	4709 (1987)	0.995
**Placebo 3% vs Adrenaline 5%**	4689 (1903)	0.996	4468 (1907)	0.996	886 (765)	1	4126 (1998)	0.994	3911 (1993)	0.995	4441 (1956)	0.995	4441 (1958)	0.996
**Placebo 4% vs Adrenaline 3%**	7343 (1375)	0.658	7210 (1534)	0.662	6439 (3023)	0.262	7303 (1422)	0.644	7422 (1295)	0.617	7186 (1574)	0.669	7241 (1504)	0.665

**Placebo 3% vs Adrenaline 4%**	7260 (1430)	0.665	7098 (1569)	0.677	1886 (2066)	0.992	6840 (1863)	0.712	6736 (1935)	0.736	7043 (1652)	0.672	7051 (1641)	0.68
**Null: Placebo 2% vs Adrenaline 2%**	7984 (242)	*0.0411*	7975 (324)	0.0462	6477 (2932)	0.3244	7957 (453)	0.0527	7965 (395)	0.0503	7963 (408)	0.0506	7964 (398)	0.0514
**Placebo 4% vs Adrenaline 2%**	4248 (1706)	1	4017 (1740)	0.999	7032 (1944)	0.884	4099 (1772)	0.999	4240 (1772)	0.999	3886 (1763)	1	3991 (1763)	1
**Placebo 2% vs Adrenaline 4%**	4019 (1674)	1	3793 (1696)	1	1053 (871)	1	3409 (1714)	1	3345 (1693)	1	3677 (1709)	1	3634 (1707)	1
**Placebo 3% vs Adrenaline 2%**	7106 (1460)	0.779	6980 (1579)	0.786	7155 (2381)	0.222	6997 (1596)	0.766	7082 (1510)	0.761	6846 (1743)	0.719	6907 (1667)	0.785
**Placebo 2% vs Adrenaline 3%**	6928 (1621)	0.789	6753 (1710)	0.799	2557 (2379)	0.984	6403 (1992)	0.826	6418 (1950)	0.841	6625 (1810)	0.806	6597 (1837)	0.811

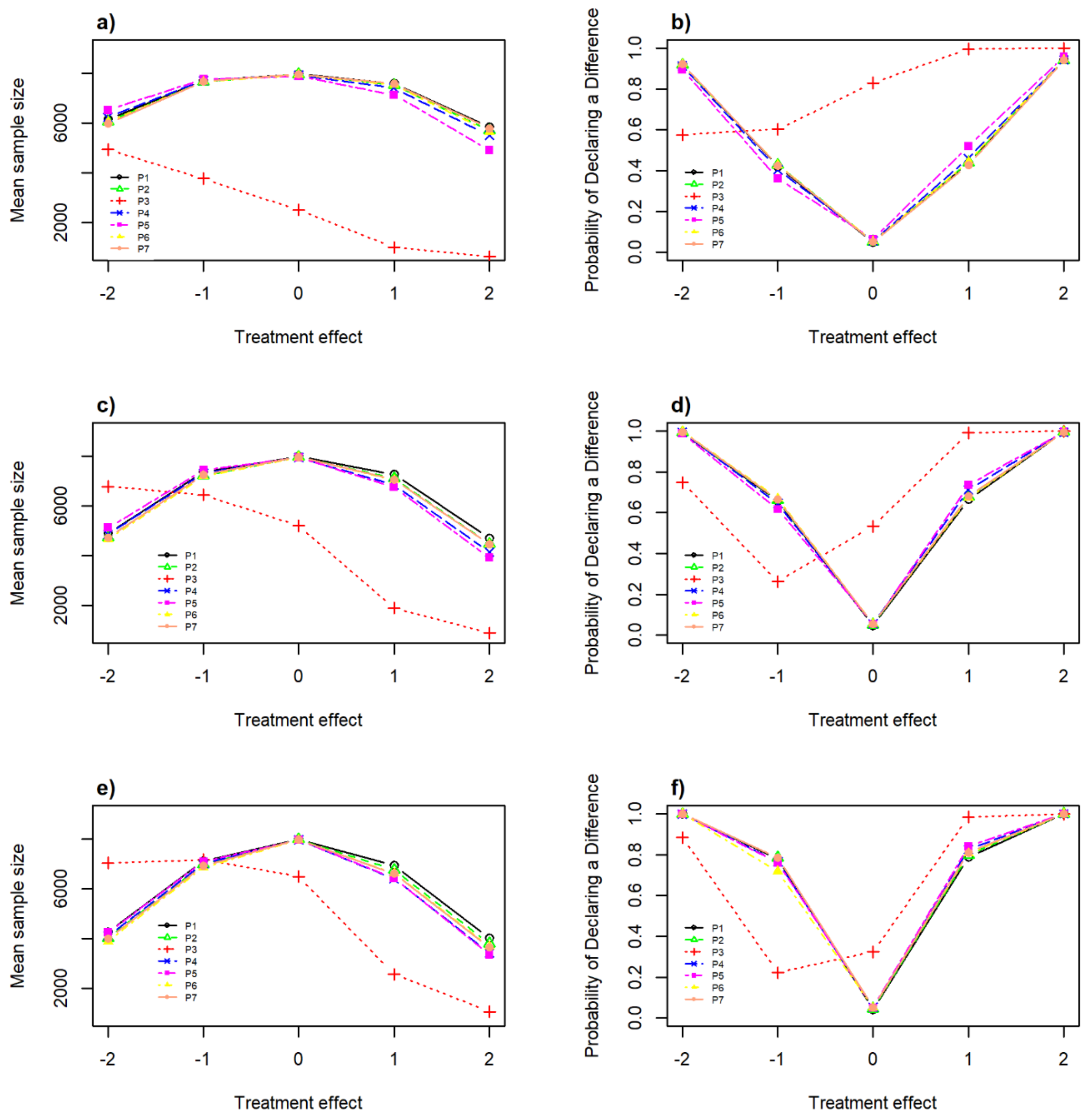

Figure A2.2. Mean sample size (left column) and probability of declaring a difference between arms (right column) for Design B2 for each prior (P1-P7) across a range of treatment effects (difference between adrenaline and placebo survival rates) and different assumed survival rates. A positive treatment effect corresponds to adrenaline being superior; a negative treatment effect corresponds to adrenaline being harmful. Figures a) and b) correspond to a control survival rate of 6%; figures c) and d) correspond to a control survival rate of 3%; figures e) and f) correspond to a control survival rate of 2%.

Table A2.6 Prior sensitivity analysis for the Bayesian designs (Design B3)

	Original prior (P1)		Clinician prior (P2)		PACA prior (P3)		PACA prior (vaguer) (P4)		RCT prior (pbo worse) (P5)		RCT prior (pbo same) (P6)		Observational studies (P7)	
	Average sample size	Prop. declaring difference	Average sample size	Prop. declaring difference	Average sample size	Prop. declaring difference	Average sample size	Prop. declaring difference	Average sample size	Prop. declaring difference	Average sample size	Prop. declaring difference	Average sample size	Prop. declaring difference
**Null: Placebo 6% vs Adrenaline 6%**	7936 (492)	*0.0515*	7917 (603)	0.0542	2929 (2995)	0.8061	7909 (635)	0.058	7871 (794)	0.0643	7912 (618)	0.0566	7918 (590)	0.0552
**Placebo 8% vs Adrenaline 6%**	5562 (1879)	0.935	5477 (1928)	0.939	5892 (3228)	0.502	5637 (1877)	0.932	5949 (1780)	0.913	5451 (1956)	0.937	5430 (1957)	0.939
**Placebo 6% vs Adrenaline 8%**	5333 (1829)	0.945	5250 (1856)	0.944	855 (619)	1	5086 (1950)	0.946	4620 (2088)	0.953	5275 (1869)	0.943	5342 (1832)	0.941
**Placebo 7% vs Adrenaline 6%**	7497 (1183)	0.424	7435 (1268)	0.443	4567 (3518)	0.505	7482 (1217)	0.417	7652 (982)	0.362	7419 (1310)	0.437	7410 (1327)	0.447
**Placebo 6% vs Adrenaline 7%**	7382 (1216)	0.435	7314 (1350)	0.442	1225 (1277)	0.993	7205 (1509)	0.457	6922 (1821)	0.509	7297 (1391)	0.436	7361 (1287)	0.432
**Null: Placebo 3% vs Adrenaline 3%**	7957 (293)	*0.0492*	7943 (440)	0.0535	5723 (3013)	0.4917	7916 (594)	0.0577	7915 (587)	0.0575	7934 (505)	0.0569	7932 (511)	0.0555
**Placebo 5% vs Adrenaline 3%**	4416 (1587)	0.995	4229 (1672)	0.995	7106 (1831)	0.732	4359 (1666)	0.997	4621 (1648)	0.994	4193 (1718)	0.995	4211 (1684)	0.996
**Placebo 3% vs Adrenaline 5%**	4186 (1535)	0.993	4023 (1599)	0.994	1083 (682)	1	3740 (1656)	0.994	3551 (1618)	0.996	3961 (1616)	0.993	3975 (1619)	0.993
**Placebo 4% vs Adrenaline 3%**	7052 (1475)	0.66	6980 (1545	0.666	7063 (2405)	0.185	7030 (1516)	0.648	7156 (1404)	0.617	6929 (1608)	0.672	6939 (1596)	0.666
**Placebo 3% vs Adrenaline 4%**	6821 (1512)	0.678	6722 (1616)	0.687	2162 (1831)	0.992	6508 (1795)	0.72	6385 (1847)	0.742	6683 (1676)	0.693	6693 (1641)	0.691
**Null: Placebo 2% vs Adrenaline 2%**	7970 (282)	*0.0415*	7963 (333)	0.0473	7003 (2245)	0.2696	7935 (484)	0.0555	7945 (430)	0.0529	7949 (415)	0.0521	7944 (439)	0.0518

**Placebo 4% vs Adrenaline 2%**	3854 (1364)	0.999	3659 (1405)	0.999	7091 (1182)	0.901	3711 (1425)	0.999	3877 (1412)	0.999	3558 (1445)	0.999	3616 (1424)	0.999
**Placebo 2% vs Adrenaline 4%**	3643 (1327)	0.999	3489 (1340)	0.999	1273 (785)	1	3143 (1383)	0.998	3109 (1365)	0.998	3385 (1374)	0.998	3374 (1389)	0.999
**Placebo 3% vs Adrenaline 2%**	6691 (1594)	0.788	6576 (1689)	0.795	7640 (1530)	0.18	6632 (1684)	0.78	6692 (1652)	0.767	6438 (1816)	0.806	6547 (1742)	0.791
**Placebo 2% vs Adrenaline 3%**	6436 (1592)	0.80	6306 (1678)	0.807	2764 (2025)	0.99	5979 (1900)	0.826	6010 (1846)	0.838	6239 (1759)	0.811	6187 (1763)	0.814

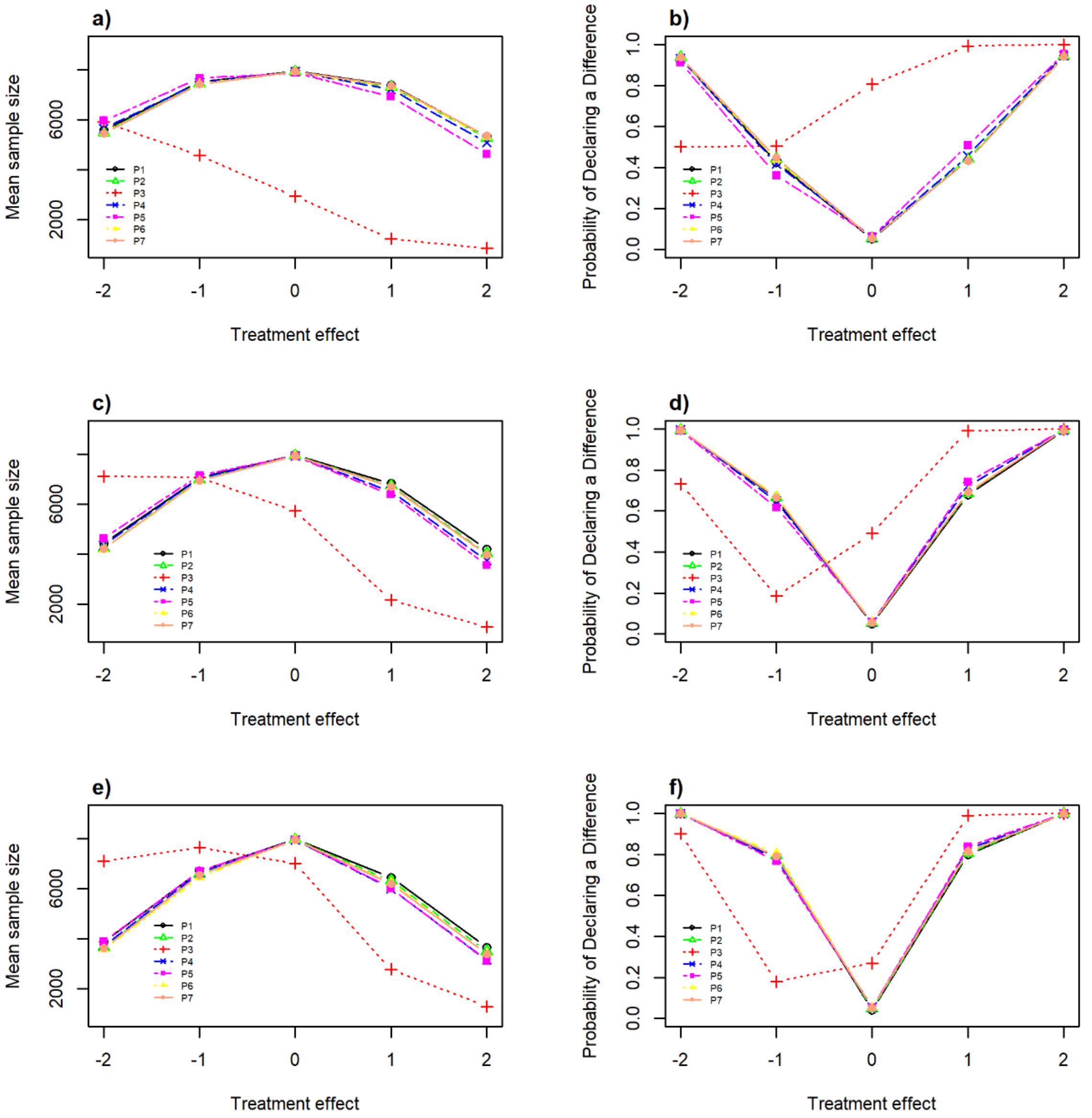

Figure A2.3. Mean sample size (left column) and probability of declaring a difference between arms (right column) for Design B3 for each prior (P1-P7) across a range of treatment effects (difference between adrenaline and placebo survival rates) and different assumed survival rates. A positive treatment effect corresponds to adrenaline being superior; a negative treatment effect corresponds to adrenaline being harmful. Figures a) and b) correspond to a control survival rate of 6%; figures c) and d) correspond to a control survival rate of 3%; figures e) and f) correspond to a control survival rate of 2%.

References

1. Jacobs IG, Finn JC, Jelinek GA, Oxer HF, Thompson PL. Effect of adrenaline on survival in out-of-hospital cardiac arrest: A randomised double-blind placebo controlled trial. Resuscitation 2011; 82:1138-43.
2. Brown CG, Martin DR, Pepe PE, Stueven H, Cummins RO, Gonzalez E, et al. A comparison of standard-dose and high-dose epinephrine in cardiac arrest outside the hospital. The Multicenter High-Dose Epinephrine Study Group. N Engl J Med. 1992; 327(15):1051-5.

3. Callaham M, Madsen CD, Barton CW, Saunders CE, Pointer J. A randomised clinical trial of high dose epinephrine and norepinephrine versus standard dose epinephrine in pre-hospital cardiac arrest. J Am Med Assoc. 1992;268:2667– 72
4. Gueugniaud PY, Mols P, Goldstein P, Pham E, Dubien PY, Deweerdt C, et al. A comparison of repeated high dose and repeated standard dose of epinephrine for cardiac arrest outside the hospital. N Engl J Med. 1998; 339:1595– 601.
5. Sherman BW, Munger MA, Foulke GE, Rutherford WF, Panacek EA. High-dose versus standard-dose epinephrine treatment of cardiac arrest after failure of standard therapy. Pharmacotherapy. 1997;17(2):242– 7.
6. Steill IG, Hebert PC, Weitzman BN, Wells GA, Raman S, Stark RM, et al. High-Dose Epinephrine in Adult Cardiac Arrest. N Engl J Med. 1992; 327:1045-1050
7. Ducros L, Vicaut E, Soleil C, Le Guen M, Gueye P, Poussant T, et al. Effect of the addition of vasopressin or vasopressin plus nitroglycerin to epinephrine on arterial blood pressure during cardiopulmonary resuscitation in humans. J Emerg Med. 2011;41(5):453–9
8. Gueugniaud PY, David JS, Chanzy E, Hubert H, DubienPY, Mauriaucourt P, et al. Vasopressin and epinephrine vs.epinephrine alone in cardiopulmonary resuscitation. N Engl J Med. 2008; 359(1):21–30.
9. Lindner KH, Dirks B, Strohmenger HU, Prengel AW,Lindner IM, Lurie KG. Randomised comparison of epinephrine and vasopressin in patients with out-of-hospital ventricular fibrillation. Lancet. 1997;349(9051):535–7
10. Ong ME, Tiah L, Leong BS, Tan EC, Ong VY, Tan EA, et al. A randomised, double-blind, multi-centre trial comparing vasopressin and adrenaline in patients with cardiac arrest presenting to or in the emergency department. Resuscitation. 2012;83:953–60
11. Wenzel V, Krismer AC, Arntz HR, Sitter H, Stadlbauer KH, Lindner KH, et al. A comparison of vasopressinand epinephrine for out-of-hospital cardiopulmonary resuscitation. N Engl J Med. 2004;350(2):105–13
12. Hagihara A, Hasegawa M, Abe T, Nagata T, Wakata Y,Miyazaki S. Prehospital epinephrine use and survival among patients with out-of-hospital cardiac arrest .JAMA. 2012;307(11):1161–8
13. Holmberg M, Holmberg S, Herlitz J. Low chance of survival among patients requiring adrenaline (epinephrine) or intubation after out-of-hospital cardiac arrest in Sweden. Resuscitation. 2002; 54(1): 37-45.
14. Kirves H, Skrifvars MB, Vähäkuopus M, Ekström K, Martikainen M, Castren M. Adherence to resuscitation guidelines during prehospital care of cardiac arrest patients. EJEM. 2007; 14(2): 75-81.
15. Olasveengen TM, Wik L, Sunde K, Steen PA. Outcome when adrenaline (epinephrine) was actually given vs. not given - post hoc analysis of a randomized clinical trial. Resuscitation. 2012; 83(3): 327-32.
16. Ong ME, Tan EH, Ng FS, Panchalingham A, Lim SH, Manning PG, et al. Survival outcomes with the introduction of intravenous epinephrine in the management of out-of-hospital cardiac arrest. Ann Emerg Med. 2007; 50(6): 635-42.
17. Vayrynen T, Kuisma M, Maatta T, Boyd J: Who survives from out-of-hospital pulseless electrical activity? Resuscitation. 2008, 76: 207-213.
18. Hayashi Y, Iwami T, Kitamura T, Nishiuchi T, Kajino K, Sakai T, et al. Impact of early intravenous epinephrine administration on outcomes following out-of-hospital cardiac arrest. Circ J. 2012; 76(7): 1639-45.
19. Herlitz J, Ekstrom L, Wennerblom B, Axelsson A, Bang A, Holmberg S. Adrenaline in out-of-hospital ventricular fibrillation. Does it make any difference? Resuscitation. 1995; 29:195–201.
