# Supplementary material for: Bayesian group sequential designs for phase III emergency medicine trials: a case study using the PARAMEDIC2 trial
Source: Trials. 2020 Jan 14;21:84. doi: 10.1186/s13063-019-4024-x (PMC6961266; doi:10.1186/s13063-019-4024-x)
Supplement: Supplementary file 3 — Additional file 3: Effect of varying recruitment rate on the operating characteristics of Design B1. [file 13063_2019_4024_MOESM3_ESM.pdf]

Additional File 3 - Effect of varying recruitment rate on the operating characteristics of Design B1

We explored the effect of having a faster recruitment rate (80/week) and a slower recruitment rate (25/week) on the operating characteristics of Bayesian design B1, which had interim analyses that occurred on a time basis. Table A3 displays these results.

The average sample size generally increased with both a faster and slower recruitment rate. The type I error was generally slightly lower with faster and slower recruitment. The power generally increased slightly with slower recruitment in the adrenaline superior scenarios; there were no overall patterns for the power using a faster recruitment rate. More early stopping occurred with a faster recruitment rate, and less occurred with a slower recruitment rate.

Table A3. Operating characteristics of Bayesian design 1 (B1), assuming faster and slower recruitment rates

	**Original recruitment (53/week)**			**Faster recruitment (80/week)**			**Slower recruitment (25/week)**		
	**Average sample size (sd)**	**Proportion stopped early (correctly)**	**Overall proportion declaring difference**	**Average sample size (sd)**	**Proportion stopped early (correctly)**	**Overall proportion declaring difference**	**Average sample size (sd)**	**Proportion stopped early (correctly)**	**Overall proportion declaring difference**
**Null: Placebo 6% vs Adrenaline 6%**	7968 (390)	0.0074	*0.0493*	7972 (363)	0.0089	*0.0486*	7964 (469)	0.0062	*0.048*
**Placebo 8% vs Adrenaline 6%**	6100 (2075)	0.502	0.935	6324 (1861)	0.632	0.935	6975 (2243)	0.175	0.933
**Placebo 6% vs Adrenaline 8%**	6019 (2107)	0.525	0.928	6200 (1967)	0.641	0.936	6773 (2405)	0.209	0.95
**Placebo 7% vs Adrenaline 6%**	7676 (1149)	0.084	0.431	7731 (956)	0.116	0.432	7831 (989)	0.029	0.383
**Placebo 6% vs Adrenaline 7%**	7654 (1174)	0.09	0.433	7727 (925)	0.13	0.409	7737 (1220)	0.045	0.474
**Null: Placebo 3% vs Adrenaline 3%**	7980 (293)	0.0053	*0.044*	7986 (250)	0.0048	*0.0429*	7985 (291)	0.0027	*0.0432*
**Placebo 5% vs Adrenaline 3%**	4842 (1985)	0.779	0.994	5144 (1796)	0.902	0.992	5953 (2803)	0.351	0.997
**Placebo 3% vs Adrenaline 5%**	4546 (1960)	0.828	0.995	5001 (1810)	0.912	0.994	5805 (2833)	0.379	0.996
**Placebo 4% vs Adrenaline 3%**	7410 (1396)	0.17	0.659	7459 (1267)	0.244	0.669	7672 (1342)	0.057	0.651
**Placebo 3% vs Adrenaline 4%**	7285 (1524)	0.202	0.663	7394 (1322)	0.276	0.65	7650 (1372)	0.062	0.688
**Null: Placebo 2% vs Adrenaline 2%**	7987 (227)	0.004	*0.0371*	7991 (181)	0.0036	*0.0373*	7995 (172)	0.001	*0.0366*
**Placebo 4% vs Adrenaline 2%**	4140 (1641)	0.908	0.999	4549 (1580)	0.968	0.9990	5439 (2917)	0.439	1
**Placebo 2% vs Adrenaline 4%**	3883 (1589)	0.934	1	4333 (1559)	0.982	1	5098 (2923)	0.5	0.999
**Placebo 3% vs Adrenaline 2%**	7172 (1587)	0.234	0.792	7215 (1359)	0.383	0.814	7631 (1397)	0.066	0.785
**Placebo 2% vs Adrenaline 3%**	6991 (1709)	0.288	0.814	7165 (1422)	0.392	0.782	7604 (1442)	0.071	0.802
