# Supplementary material for: Bayesian group sequential designs for phase III emergency medicine trials: a case study using the PARAMEDIC2 trial
Source: Trials. 2020 Jan 14;21:84. doi: 10.1186/s13063-019-4024-x (PMC6961266; doi:10.1186/s13063-019-4024-x)
Supplement: Supplementary file 4 — Additional file 4: Operating characteristics for Bayesian design based on frequentist stopping boundaries. [file 13063_2019_4024_MOESM4_ESM.pdf]

Additional File 4 - Operating characteristics for Bayesian design based on frequentist stopping boundaries

The stopping boundaries for this design were obtained from 1 – nominal p-values obtained from the frequentist stopping boundaries in Additional File 1 Table A1.1. Whilst this design had smaller average sample sizes compared to Bayesian Designs B1-B3, it often had smaller probabilities of declaring a difference between the arms particularly when adrenaline was assumed to be superior. This design also often gave a high proportion of flip-flops, particularly for the "adrenaline superior" scenarios. We therefore used stricter stopping boundaries for Designs B1-B3.

Table A4. Operating characteristics for Bayesian design based on original frequentist stopping boundaries for PARAMEDIC2

Design and scenarios[a]	Average duration (weeks)	Average sample size (sd)	Proportion stopped early[b]	Overall proportion declaring a difference[c]	Proportion that did not declare a difference	Proportion flip-flop [d]	Average probability adrenaline superior
Null: Placebo 6% vs Adrenaline 6%	166	7836 (862)	0.0307	*0.0414*	0.9488	0.0098	0.5064
Placebo 8% vs Adrenaline 6%	111	4923 (1882)	0.782	0.921	0.073	0.006	0.0078
Placebo 6% vs Adrenaline 8%	108	4778 (2348)	0.631	0.777	0.134	0.089	0.9919
Placebo 7% vs Adrenaline 6%	150	7019 (1612)	0.283	0.437	0.548	0.015	0.0996
Placebo 6% vs Adrenaline 7%	151	7077 (1843)	0.185	0.262	0.695	0.043	0.8992
Null: Placebo 3% vs Adrenaline 3%	166	7881 (706)	0.024	*0.036*	0.9565	0.0075	0.5033
Placebo 5% vs Adrenaline 3%	91	3889 (1402)	0.948	0.991	0.006	0.003	0.0021
Placebo 3% vs Adrenaline 5%	84	3536 (1804)	0.844	0.908	0.013	0.079	0.997
Placebo 4% vs Adrenaline 3%	138	6384 (1856)	0.461	0.649	0.34	0.011	0.0425
Placebo 3% vs Adrenaline 4%	140	6486 (2130)	0.319	0.473	0.472	0.055	0.9559
Null: Placebo 2% vs Adrenaline 2%	167	7929 (523)	0.0163	*0.0292*	0.9964	0.0044	0.5026
Placebo 4% vs Adrenaline 2%	83	3472 (1100)	0.985	0.999	0.001	0	0.0012
Placebo 2% vs Adrenaline 4%	75	3029 (1424)	0.911	0.938	0.003	0.059	0.9978
Placebo 3% vs Adrenaline 2%	132	6043 (1870)	0.563	0.778	0.216	0.006	0.0238
Placebo 2% vs Adrenaline 3%	131	6017 (2211)	0.429	0.505	0.338	0.066	0.9761

[a]Different effect size scenarios that were simulated for each design are given as placebo 30-day survival rate vs adrenaline rate. [b]Proportion of simulations that stopped early and were declared to have a difference (in the correct direction) at the final analysis. [c]The simulated type I errors are italicised. [d]These simulations were stopped early for efficacy or harm, but did not meet the critical values to declare a difference between the treatments at the final analysis once all patients were followed up (insufficient evidence of a difference)
