# Supplementary material for: Bayesian group sequential designs for phase III emergency medicine trials: a case study using the PARAMEDIC2 trial
Source: Trials. 2020 Jan 14;21:84. doi: 10.1186/s13063-019-4024-x (PMC6961266; doi:10.1186/s13063-019-4024-x)
Supplement: Supplementary file 5 — Additional file 5: Example trials. [file 13063_2019_4024_MOESM5_ESM.pdf]

Additional File 5 - Example trials

Here we present single trial simulations for each Bayesian design to illustrate the decisions that may be made at each analysis based on the accumulated data. The true 30-day survival rates are assumed to be 2% and 3% for the placebo and adrenaline arm, respectively. Prior P1 was used.

A5.1 Bayesian Design B1

Table A5.1.1 Example trial 1: late declaration of adrenaline superior for Bayesian Design B1

Analysis #	Num subjects recruited (pbo:adr)	Num subjects with complete data (pbo:adr)	Pr(adrenaline superior)	Pr(placebo superior)	Duration (weeks)	Posterior Estimated response pbo	Posterior Estimated response adr
**1**	50 (25:25)	5: 4	0.3472	0.6528	8.33206	0.080217	0.063581
**2**	463 (231:232)	142:142	0.7604	0.2396	21.3321	0.029805	0.041469
**3**	1092 (546:546)	439: 439	0.6692	0.3308	34.3321	0.02723	0.031666
**4**	1802 (901:901)	787: 788	0.7264	0.2736	47.3321	0.025329	0.029781
**5**	2491 (1245:1246)	1131:1130	0.5792	0.4208	60.3321	0.026321	0.027568
**6**	3170 (1585:1585)	1479:1479	0.7412	0.2588	73.3321	0.026597	0.03042
**7**	3858 (1929:1929)	1811:1810	0.7776	0.2224	86.3321	0.024692	0.02865
**8**	4518 (2259:2259)	2153:2152	0.7688	0.2312	99.3321	0.024297	0.027893
**9**	5199 (2600:2599)	2491:2492	0.8224	0.1776	112.332	0.024072	0.028357
**10**	5889 (2945:2944)	2828:2829	0.9664	0.0336	125.332	0.022784	0.030505
**Final**	8000 (4000:4000)	4000:4000	0.9816	0.0184	168.669	0.022815	0.03073

Pbo = Placebo; Adr = Adrenaline

Table A5.1.2 Example trial 2: correct early stopping for adrenaline superior for Bayesian Design B1

Analysis #	Num subjects recruited (pbo:adr)	Num subjects with complete data (pbo:adr)	Pr(adrenaline superior)	Pr(placebo superior)	Duration (weeks)	Posterior Estimated response pbo	Posterior Estimated response adr
**1**	50 (25:25)	4: 4	0.4972	0.5028	8.9265	0.065081	0.065374
**2**	428 (214:214)	130:130	0.838	0.162	21.9265	0.031838	0.048759
**3**	1131 (566:565)	454 : 454	0.9032	0.0968	34.9265	0.021535	0.034755
**4**	1846 (923:923)	802 : 803	0.9568	0.0432	47.9265	0.021632	0.034661
**5**	2508 (1254:1254)	1139 : 1139	0.9904	0.0096	60.9265	0.020487	0.035433
**6**	3222 (1611:1611)	1487 : 1486	1	0	73.9265	0.01828	0.038646
**Final**	3222 (1611:1611)	1611 : 1611	0.9992	0.0008	78.2033	0.020416	0.039784

Pbo = Placebo; Adr = Adrenaline

Table A5.1.3 Example trial 3: late futility (unable to declare either arm superior) for Bayesian Design B1

Analysis #	Num subjects recruited (pbo:adr)	Num subjects with complete data (pbo:adr)	Pr(adrenaline superior)	Pr(placebo superior)	Duration (weeks)	Posterior Estimated response pbo	Posterior Estimated response adr
**1**	50 (25:25)	2 : 3	0.5124	0.4876	7.40074	0.06531	0.065537
**2**	403 (201:202)	109 : 108	0.5048	0.4952	20.4007	0.044058	0.044921
**3**	1015 (508:507)	399 : 398	0.8088	0.1912	33.4007	0.025265	0.034331
**4**	1664 (832:832)	720 : 720	0.936	0.064	46.4007	0.024659	0.036738
**5**	2331 (1166:1165)	1051 : 1051	0.958	0.042	59.4007	0.019243	0.02995
**6**	3040 (1520:1520)	1403: 1402	0.99	0.01	72.4007	0.019531	0.031902
**7**	3721 (1860:1861)	1757 : 1757	0.9684	0.0316	85.4007	0.022297	0.032021
**8**	4401 (2200:2201)	2085 : 2085	0.9896	0.0104	98.4007	0.021315	0.033305
**9**	5145 (2573:2572)	2462 : 2462	0.9828	0.0172	111.401	0.022457	0.032028
**10**	5868 (2934:2934)	2822 : 2821	0.9816	0.0184	124.401	0.022476	0.031262

**Final**	8000 (4000:4000)	4000 : 4000	0.97	0.03	169.469	0.022668	0.029945

Pbo = Placebo; Adr = Adrenaline

A5.2 Bayesian Design B2

Table A5.2.1 Example trial 1: late declaration of adrenaline superior for Bayesian Design B2

Analysis #	Num subjects recruited (pbo:adr)	Num subjects with complete data (pbo:adr)	Pr(adrenaline superior)	Pr(placebo superior)	Duration (weeks)	Posterior Estimated response pbo	Posterior Estimated response adr
**1**	50 (25:25)	5 : 4	0.6544	0.3456	7.41905	0.082909	0.030122
**2**	300 (150:150)	87 : 87	0.8352	0.1648	17.861	0.068242	0.015621
**3**	600 (300:300)	202 : 202	0.8736	0.1264	24.839	0.04608	0.008961
**4**	1000 (500:500)	382 : 383	0.8224	0.1776	32.5322	0.041917	0.007709
**5**	1450 (725:725)	611 : 610	0.7612	0.2388	41.112	0.036515	0.006451
**6**	1900 (950:950)	842 : 842	0.9204	0.0796	49.6368	0.034991	0.004876
**7**	2650 (1325:1325)	1211 : 1211	0.9472	0.0528	64.2866	0.035309	0.004183
**8**	3650 (1825:1825)	1686 : 1687	0.986	0.014	82.4477	0.033567	0.003417
**9**	5000 (2500:2500)	2399 : 2399	0.9804	0.0196	107.912	0.028619	0.002645
**10**	6500 (3250:3250)	3139 : 3139	0.9836	0.0164	134.781	0.028702	0.00239
**11**	7000 (3500:3500)	3397 : 3396	0.9844	0.0156	144.551	0.027395	0.002389
**12**	7500 (3750:3750)	3638 : 3638	0.9988	0.0012	153.349	0.028662	0.002199
**Final**	8000 (4000:4000)	4000 : 4000	0.9968	0.0032	166.774	0.029923	0.002219

Pbo = Placebo; Adr = Adrenaline

Table A5.2.2 Example trial 2 – correct early stopping for adrenaline superior for Bayesian Design B2

Analysis #	Num subjects recruited (pbo:adr)	Num subjects with complete data (pbo:adr)	Pr(adrenaline superior)	Pr(placebo superior)	Duration (weeks)	Posterior Estimated response pbo	Posterior Estimated response adr
**1**	50 (25:25)	7 : 6	0.5072	0.4928	7.82915	0.077761	0.078654
**2**	300 (150:150)	84 : 83	0.7404	0.2596	17.2108	0.042907	0.057394
**3**	600 (300:300)	200 : 200	0.6496	0.3504	23.9969	0.033909	0.039471
**4**	1000 (500:500)	383 : 383	0.7652	0.2348	31.4113	0.029442	0.037607
**5**	1450 (725:725)	613 : 614	0.7212	0.2788	40.3846	0.028569	0.034103
**6**	1900 (950:950)	828 : 828	0.9312	0.0688	48.6892	0.024418	0.03606
**7**	2650 (1325:1325)	1217 : 1217	0.9984	0.0016	62.5166	0.021419	0.040361
**8**	3650 (1825:1825)	1713 : 1713	1	0	81.0606	0.020502	0.039742
**Final**	3650 (1825:1825)	1825 : 1825	1	0	85.3506	0.020537	0.039308

Pbo = Placebo; Adr = Adrenaline

Table A5.2.3 Example trial 3 – late futility (unable to declare either arm superior) for Bayesian Design B2

Analysis #	Num subjects recruited (pbo:adr)	Num subjects with complete data (pbo:adr)	Pr(adrenaline superior)	Pr(placebo superior)	Duration (weeks)	Posterior Estimated response pbo	Posterior Estimated response adr
**1**	50 (25:25)	5 : 4	0.3472	0.6528	8.33206	0.080217	0.063581
**2**	300 (150:150)	82 : 82	0.6212	0.3788	17.6843	0.038318	0.045343
**3**	600 (300:300)	189 : 190	0.6816	0.3184	24.182	0.028795	0.035579
**4**	1000 (500:500)	391 : 392	0.6504	0.3496	32.1864	0.030224	0.034617
**5**	1450 (725:725)	604 : 604	0.746	0.254	40.6421	0.028443	0.034405
**6**	1900 (950:950)	838 : 838	0.782	0.218	49.288	0.025364	0.031048
**7**	2650 (1325:1325)	1212 : 1212	0.868	0.132	63.3281	0.02398	0.031243
**8**	3650 (1825:1825)	1705 : 1705	0.8428	0.1572	82.5533	0.023734	0.029024
**9**	5000 (2500:2500)	2375 : 2375	0.8716	0.1284	108.341	0.024081	0.029051
**10**	6500 (3250:3250)	3136 : 3137	0.8232	0.1768	136.247	0.023049	0.026732
**11**	7000 (3500:3500)	3387 : 3386	0.772	0.228	146.008	0.023744	0.026343

**12**	7500 (3750:3750)	3629 : 3630	0.7648	0.2352	155.76	0.024624	0.027192
**Final**	8000 (4000:4000)	4000 : 4000	0.59	0.41	168.669	0.025522	0.026439

Pbo = Placebo; Adr = Adrenaline

A5.3 Bayesian Design B3

Table A5.3.1 Example trial 1 – late declaration of adrenaline superior for Bayesian Design B3

Analysis #	Num subjects recruited (pbo:adr)	Num subjects with complete data (pbo:adr)	Pr(adrenaline superior)	Pr(placebo superior)	Duration (weeks)	Posterior Estimated response pbo	Posterior Estimated response adr
**1**	500 (250:250)	169 : 170	0.5124	0.4876	22.7785	0.044554	0.044855
**2**	1000 (500:500)	376 : 377	0.526	0.474	32.221	0.032582	0.033697
**3**	1500 (750:750)	639 : 640	0.9144	0.0856	41.9967	0.022061	0.033837
**4**	2000 (1000:1000)	880 : 879	0.7744	0.2256	50.925	0.022876	0.027999
**5**	2500 (1250:1250)	1121 : 1121	0.8528	0.1472	59.686	0.020182	0.026063
**6**	3000 (1500:1500)	1393 : 1394	0.9372	0.0628	69.2772	0.016812	0.024391
**7**	3500 (1750:1750)	1635 : 1634	0.9696	0.0304	78.7475	0.01736	0.025919
**8**	4000 (2000:2000)	1891 : 1891	0.9808	0.0192	88.7248	0.018502	0.028236
**9**	4500 (2250:2250)	2124 : 2123	0.9924	0.0076	97.3334	0.018896	0.029434
**10**	5000 (2500:2500)	2385 : 2384	0.9904	0.0096	106.15	0.019186	0.028967
**11**	5500 (2750:2750)	2625 : 2624	0.9896	0.0104	115.093	0.018792	0.027503
**12**	6000 (3000:3000)	2885 : 2885	0.9844	0.0156	124.507	0.019165	0.027627
**13**	6500 (3250:3250)	3135 va 3135	0.9784	0.0216	133.758	0.020962	0.028579
**14**	7000 (3500:3500)	3380 : 3381	0.9584	0.0416	142.705	0.022185	0.028905
**15**	7500 (3750:3750)	3622 : 3622	0.9644	0.0356	152.044	0.021798	0.02852
**Final**	8000 (4000:4000)	4000 : 4000	0.988	0.012	166.359	0.021272	0.028163

Pbo = Placebo; Adr = Adrenaline

Table A5.3.2 Example trial 2 – correct early stopping for adrenaline superior for Bayesian Design B3

Analysis #	Num subjects recruited (pbo:adr)	Num subjects with complete data (pbo:adr)	Pr(adrenaline superior)	Pr(placebo superior)	Duration (weeks)	Posterior Estimated response pbo	Posterior Estimated response adr
**1**	500 (250:250)	164 : 163	0.6056	0.3944	22.3558	0.03447	0.038559
**2**	1000 (500:500)	380 : 380	0.6432	0.3568	31.6764	0.0277	0.032008
**3**	1500 (750:750)	637 : 637	0.8168	0.1832	40.9482	0.025318	0.033527
**4**	2000 (1000:1000)	883 : 882	0.972	0.028	51.0157	0.023852	0.03819
**5**	2500 (1250:1250)	1135 : 1135	0.9816	0.0184	60.2265	0.020758	0.033876
**6**	3000 (1500:1500)	1380 : 1381	0.996	0.004	68.9814	0.018799	0.034846
**7**	3500 (1750:1750)	1625 : 1625	1	0	78.6378	0.018472	0.033847
**Final**	3500 (1750:1750)	1750 : 1750	0.9876	0.0124	82.9278	0.020841	0.032612

Pbo = Placebo; Adr = Adrenaline

Table A5.3.3 Example trial 3 – late futility (unable to declare either arm superior) for Bayesian Design B3

Analysis #	Num subjects recruited (pbo:adr)	Num subjects with complete data (pbo:adr)	Pr(adrenaline superior)	Pr(placebo superior)	Duration (weeks)	Posterior Estimated response pbo	Posterior Estimated response adr
**1**	500 (250:250)	151 : 151	0.2632	0.7368	22.2181	0.052038	0.040395
**2**	1000 (500:500)	389 : 389	0.6476	0.3524	32.5007	0.040098	0.04472
**3**	1500 (750:750)	644 : 644	0.5236	0.4764	42.3625	0.039668	0.040303
**4**	2000 (1000:1000)	896 : 896	0.468	0.532	53.149	0.0378	0.037029
**5**	2500 (1250:1250)	1132 : 1132	0.5792	0.4208	61.849	0.033451	0.034759
**6**	3000 (1500:1500)	1389 : 1388	0.7532	0.2468	70.9108	0.02901	0.033294
**7**	3500 (1750:1750)	1638 : 1638	0.892	0.108	80.4398	0.025482	0.032316
**8**	4000 (2000:2000)	1883 : 1883	0.9244	0.0756	89.399	0.024279	0.031103
**9**	4500 (2250:2250)	2127 : 2127	0.8884	0.1116	98.1731	0.024034	0.029843

**10**	5000 (2500:2500)	2384 : 2385	0.892	0.108	107.394	0.022914	0.02822
**11**	5500 (2750:2750)	2635 : 2635	0.8428	0.1572	116.949	0.023261	0.02732
**12**	6000 (3000:3000)	2876 : 2876	0.9116	0.0884	126.103	0.022945	0.028472
**13**	6500 (3250:3250)	3131 : 3132	0.8436	0.1564	135.015	0.024026	0.027825
**14**	7000 (3500:3500)	3383 : 3384	0.8576	0.1424	144.23	0.024177	0.028346
**15**	7500 (3750:3750)	3630 : 3630	0.8212	0.1788	153.678	0.024223	0.027696
**Final**	8000 (4000:4000)	4000 : 4000	0.9568	0.0432	166.868	0.023363	0.029361

Pbo = Placebo; Adr = Adrenaline
