# Supplementary material for: Bayesian group sequential designs for phase III emergency medicine trials: a case study using the PARAMEDIC2 trial
Source: Trials. 2020 Jan 14;21:84. doi: 10.1186/s13063-019-4024-x (PMC6961266; doi:10.1186/s13063-019-4024-x)
Supplement: Supplementary file 6 — Additional file 6: Re-executing PARAMEDIC2 with Bayesian group sequential designs. [file 13063_2019_4024_MOESM6_ESM.pdf]

Additional File 6 - Re-executing PARAMEDIC2 with Bayesian group sequential designs

Tables A6.1-A6.3 present the results of the virtual execution of the PARAMEDIC2 trial using Bayesian designs B1-B3 and prior P1.

Table A6.1 Re-execution of PARAMEDIC2 trial using Bayesian group sequential design B1[a]

Interim	Number of subjects recruited (pbo:adr)	Primary outcome pbo (%) vs adr (%)	Posterior Estimated response pbo (95% CrI)	Posterior Estimated response adr (95% CrI)	Posterior Probability adr superior
**1**	44 (30:14)	0/29 (0%) vs 1/13 (7.7%)	4.9% (2%, 9.7%)	7.0% (2.5%, 4.3%)	0.7288
**2**	97 (58:39)	0/55 (0%) vs 1/35 (2.9%)	4% (1.7%, 7.6%)	5.4% (2.1%, 0.6%)	0.705
**3**	245 (134:111)	0/133 (0%) vs 2/110 (1.8%)	2.8% (1.3%, 5.2%)	3.8% (1.8%, 6.8%)	0.7538
**4**	665 (332:333)	6/320 (1.9%) vs 4/320 (1.3%)	2.9% (1.6%, 4.8%)	2.5% (1.3%, 4.0%)	0.3414
**5**	1226 (595: 631)	9/582 (1.5%) vs 9/610 (1.5%)	2.3% (1.4%, 3.4%)	2.2% (1.3%, 3.4%)	0.4514
**6**	2008 (993:1015)	15/984 (1.5%) vs 20/1002 (2%)	2.0% (1.3%, 2.8%)	2.4% (1.6%, 3.4%)	0.7382
**7**	2785 (1388:1397)	23/1378 (1.7%) vs 32/1379 (2.3%)	2% (1.4%, 2.7%)	2.6% (1.8%, 3.4%)	0.8522
**8**	3551 (1757:1794)	31/1741 (1.8%) vs 49/1772 (2.8%)	2% (1.5%, 2.7%)	2.9% (2.2%, 3.7%)	0.9588
**9**	4737 (2358:2379)	41/2336 (1.8%) vs 69/2355 (2.9%)	1.9% (1.5%, 2.6%)	3.1% (2.4%, 3.7%)	0.9958
**10**	6018 (3006:3012)	55/2829 (1.9%) vs 85/2804(3.0%)	2.1% (1.6%, 2.6%)	3.2% (2.5%, 3.8%)	0.9906
**Final analysis**	8014 (3999:4015)	94/3995 (2.4%) vs 130/4012 (3.2%)	2.5% (2%, 2.9%)	3.3% (2.7%, 3.9%)	0.9878

[a] The re-execution was performed using prior P1; 95% CrI = 95% Credible interval. Pbo = Placebo; Adr = Adrenaline.

Table A6.2 Re-execution of PARAMEDIC2 trial using Bayesian group sequential design B2 [a]

Interim	Number of subjects recruited (pbo:adr)	Primary outcome pbo (%) vs adr (%)	Posterior Estimated response pbo (95% CrI)	Posterior Estimated response adr (95% CrI)	Posterior Probability adr superior
**1**	50 (33:17)	0/8 (0%) vs 1/3 (33.3%)	6.3% (2.4%, 13.8%)	8.2% (3.0%, 17.3%)	0.6594
**2**	300 (161:139)	3/114 (2.6%) vs 4/95 (4.2%)	4.3% (2.1%, 7.5%)	5.3% (2.5%, 8.9%)	0.6738
**3**	600 (304:296)	5/195 (2.6%) vs 5/187 (2.7%)	3.8% (2.0%, 6.3%)	3.8% (2.1%, 6.2%)	0.523
**4**	1000 (479:521)	10/362 (2.8%) vs 9/368 (2.4%)	3.5% (2.1%, 5.2%)	3.3% (1.9%, 5.0%)	0.411
**5**	1450 (703:747)	12/520 (2.3%) vs 14/573 (2.4%)	2.9% (1.8%, 4.4%)	3.0% (1.9%, 4.3%)	0.539
**6**	1900 (940:960)	17/733 (2.3%) vs 22/778 (2.8%)	2.8% (1.7%, 4.0%)	3.2% (2.2%, 4.4%)	0.6988
**7**	2650 (1325: 1325)	24/1147 (2.1%) vs 31/1161 (2.7%)	2.5% (1.7%, 3.4%)	2.9% (2.1%, 3.9%)	0.787
**8**	3650 (1807:1843)	30/1638 (1.8%) vs 52/1641 (3.2%)	2.1% (1.5%, 2.8%)	3.3% (2.5%, 4.2%)	0.9848
**9**	5000 (2489:2511)	46/2157 (2.1%) vs 72/2195 (3.3%)	2.3% (1.7%, 3.0%)	3.4% (2.7%, 4.1%)	0.984
**10**	6500 (3235: 3265)	61/2991 (2.0%) vs 94/3000 (3.1%)	2.2% (1.7%, 2.7%)	3.2% (2.6%, 3.9%)	0.9968
**11**	7000 (3490:3510)	65/3273 (2%) vs 104/3288 (3.2%)	2.1% (1.7%, 2.6%)	3.2% (2.7%, 3.9%)	**0.9988**
**12**	NA	NA	NA	NA	NA
**Final analysis**	7000 (3490: 3510)	71/3487 (2.0%) vs 112/3507 (3.2%)	2.2% (1.8%, 2.7%)	3.3% (2.7%, 3.9%)	0.9974

[a] The re-execution was performed using prior P1; 95% CrI = 95% Credible interval. Pbo = Placebo; Adr = Adrenaline.

Table A6.3 Re-execution of PARAMEDIC2 trial using Bayesian group sequential design B3 [a]

Interim	Number of subjects recruited (pbo:adr)	Primary outcome pbo (%) vs adr (%)	Posterior Estimated response pbo (95% CrI)	Posterior Estimated response adr (95% CrI)	Posterior Probability adr superior
**1**	500 (253:247)	5/162 (3.1%) vs 4/140 (2.9%)	4.3% (2.3%, 7.1%)	4.2% (2.2%, 7.0%)	0.4726
**2**	1000 (479:521)	10/362 (2.8%) vs 9/368 (2.4%)	3.5% (2.1%, 5.2%)	3.3% (1.9%, 5.0%)	0.411
**3**	1500 (727: 773)	13/549 (2.4%) vs 15/601 (2.5%)	3.0% (1.8%, 4.5%)	3.0% (2.0%, 4.4%)	0.5216
**4**	2000 (989:1011)	17/794 (1.7%) vs 22/829 (2.7%)	2.6% (1.7%, 3.7%)	3.0% (2.0%, 4.2%)	0.714
**5**	2500 (1246: 1254)	21/1065 (2.0%) vs 28/1076 (2.6%)	2.3% (1.6%, 3.3%)	2.9% (2.0%, 4.0%)	0.8126
**6**	3000 (1491: 1509)	26/1325 (2.0%) vs 35/1332 (2.6%)	2.3% (1.6%, 3.1%)	2.9% (2.1%, 3.8%)	0.8502
**7**	3500 (1740:1760)	30/1576 (1.9%) vs 47/1581 (3.0%)	2.2% (1.6%, 2.9%)	3.2% (2.4%, 4.1%)	0.9572
**8**	4000 (1986: 2014)	33/1745 (1.9%) vs 56/1772 (3.2%)	2.1% (1.5%, 2.9%)	3.3% (2.6%, 4.1%)	0.9858
**9**	4500 (2227: 2273)	41/1957 (2.1%) vs 65/1995 (3.3%)	2.3% (1.7%, 3.0%)	3.4% (2.6%, 4.2%)	0.9834
**10**	5000 (2489:2511)	46/2157 (2.1%) vs 72/2195 (3.3%)	2.3% (1.7%, 3.0%)	3.4% (2.7%, 4.1%)	0.984
**11**	5500 (2764: 2736)	51/2441 (2.1%) vs 82/2463 (3.3%)	2.3% (1.7%, 2.8%)	3.4% (2.8%, 4.2%)	0.9954
**12**	6000 (2996: 3004)	56/2750 (2.0%) vs 87/2726 (3.2%)	2.2% (1.7%, 2.8%)	3.3% (2.7%, 4.0%)	0.993
**13**	6500 (3235: 3265)	61/2991 (2.0%) vs 94/3000 (3.1%)	2.2% (1.7%, 2.7%)	3.2% (2.6%, 3.9%)	**0.9968**
**14**	NA	NA	NA	NA	NA
**15**	NA	NA	NA	NA	NA
**Final analysis**	6500 (3235:3265)	65/3232 (2.0%) vs 102/3262 (3.1%)	2.2% (1.7%, 2.7%)	3.2% (2.6%, 3.8%)	0.996

[a] The re-execution was performed using prior P1; 95% CrI = 95% Credible interval. Pbo = Placebo; Adr = Adrenaline.

Prior sensitivity of virtual re-executions

Tables A6.4-A6.6 present a prior sensitivity analysis for Bayesian Designs B1-B3 using all seven priors mentioned in Additional File 2.

Table A6.4 Re-execution of PARAMEDIC2 trial showing the posterior probability that adrenaline is superior, using Bayesian group sequential design B1 with different priors

	Prior						
**Interim**	**P1**	**P2**	**P3**	**P4**	**P5**	**P6**	**P7**
**1**	0.7288	0.7114	0.999	0.8924	0.869	0.781	0.8074
**2**	0.705	0.6942	0.9998	0.8692	0.8124	0.7522	0.8038
**3**	0.7538	0.7578	0.9988	0.9014	0.7962	0.8006	0.8768
**4**	0.3414	0.3208	0.985	0.4148	0.3636	0.3036	0.353
**5**	0.4514	0.4526	0.9732	0.5656	0.498	0.4664	0.5142
**6**	0.7382	0.7576	0.99	0.816	0.798	0.7684	0.8084
**7**	0.8522	0.8774	0.9948	0.9036	0.9008	0.8734	0.8996
**8**	0.9588	0.9674	**0.9998**	0.9812	0.979	0.9716	0.9736
**9**	0.9958	0.9974	NA	0.9982	0.997	0.9954	0.997
**10**	0.9906	0.9914	NA	0.9928	0.9932	0.9936	0.9946
**Final analysis**	0.9878	0.9854	0.9998	0.9924	0.9934	0.9916	0.9912

When the PACA prior (P3) was used during the virtual re-executions, Bayesian design B1 recommended stopping the trial at interim analysis 8 after 3551 patients had been recruited. All other priors did not stop the trial early but declared adrenaline superior at the final analysis. The PACA prior (P3) was strongly informative and so caution should be taken when interpreting these results.

Table A6.5 Re-execution of PARAMEDIC2 trial showing the posterior probability that adrenaline is superior, using Bayesian group sequential design B2 with different priors

	Prior						
**Interim**	**P1**	**P2**	**P3**	**P4**	**P5**	**P6**	**P7**
**1**	0.6594	0.6668	0.9988	0.8734	0.8926	0.7188	0.6784
**2**	0.6738	0.671	0.9994	0.8106	0.8296	0.7052	0.7202
**3**	0.523	0.516	0.9972	0.6336	0.6656	0.5336	0.5582
**4**	0.411	0.4072	0.9898	0.5058	0.526	0.3898	0.4252
**5**	0.539	0.5456	0.994	0.6414	0.64	0.5534	0.5732
**6**	0.6988	0.7208	0.996	0.7956	0.7972	0.7322	0.7338
**7**	0.787	0.8004	0.9942	0.855	0.8552	0.8162	0.824
**8**	0.9848	0.9904	**1**	0.9926	0.991	0.9864	0.9916
**9**	0.984	0.9876	NA	0.9938	0.9912	0.9866	0.9886
**10**	0.9968	0.9968	NA	0.9982	0.9982	0.9962	0.9962
**11**	**0.9988**	**0.9994**	NA	**0.9996**	**0.9998**	**0.9996**	**0.999**
**12**	NA	NA	NA	NA	NA		
**Final analysis**	0.9974	0.9988	0.9996	0.9996	0.9988	0.998	0.9984

When the PACA prior (P3) was used during the virtual re-executions, Bayesian design B2 recommended stopping the trial at interim analysis 8 after 3650 patients had been recruited. All

other priors recommended stopping the trial early at interim analysis 11 after 7000 patients had been recruited. Adrenaline was declared superior at the final analysis for all priors.

Table A6.6 Re-execution of PARAMEDIC2 trial showing the posterior probability that adrenaline is superior, using Bayesian group sequential design B3 with different priors

				Prior			
**Interim**	**P1**	**P2**	**P3**	**P4**	**P5**	**P6**	**P7**
**1**	0.4726	0.4496	0.997	0.5876	0.6274	0.4566	0.4754
**2**	0.411	0.4072	0.9898	0.5058	0.526	0.3898	0.4252
**3**	0.5216	0.5582	0.991	0.6208	0.648	0.5618	0.5796
**4**	0.714	0.7424	0.9938	0.7968	0.7964	0.7408	0.7506
**5**	0.8126	0.8276	0.9982	0.8684	0.8716	0.8428	0.8542
**6**	0.8502	0.8666	0.998	0.9072	0.896	0.8748	0.8844
**7**	0.9572	0.965	0.9992	0.9732	0.978	0.9746	0.967
**8**	0.9858	0.9902	**0.9998**	0.9934	0.9948	0.9938	0.9916
**9**	0.9834	0.987	NA	0.9992	0.9916	0.9886	0.988
**10**	0.984	0.9876	NA	0.9938	0.9912	0.9866	0.9886
**11**	0.9954	0.995	NA	0.9966	0.9976	0.9966	0.9968
**12**	0.993	0.9936	NA	**0.9976**	0.996	0.9958	**0.9966**
**13**	**0.9968**	**0.9968**	NA	NA	**0.9982**	**0.9962**	NA
**14**	NA	NA	NA	NA	NA	NA	NA
**15**	NA	NA	NA	NA	NA	NA	NA
**Final analysis**	0.996	0.9952	0.9992	0.9986	0.9972	0.995	0.9984

When priors P1, P2, P5 and P6 were used, the trial stopped early at interim analysis 13 when 6500 patients had been recruited. When priors P4 and P7 were used, the trial stopped early at interim analysis 12 when 6000 patients had been recruited. When prior P3 was used, the trial stopped early at interim analysis 8 when 4000 patients had been recruited. Adrenaline was declared superior at the final analysis under each of the priors.
